# Catchment structure and functioning mediates beaver activity in contrasting lowland agricultural landscapes

**DOI:** 10.1002/eap.70335

**Published:** 2026-10-26

**Authors:** Maria Magdalena Warter, Dörthe Tetzlaff, Tobias Goldhammer, Luisa Wurbs, Lisa Grof, Franziska Schmid, Chris Soulsby

**Affiliations:** ^1^ Department of Ecohydrology and Biogeochemistry Leibniz Institute of Freshwater Ecology and Inland Fisheries Berlin Germany; ^2^ Northern Rivers Institute University of Aberdeen, St. Mary's Building, Kings College Old Aberdeen Scotland UK; ^3^ Department of Geography Humboldt University of Berlin Berlin Germany

**Keywords:** climate resilience, ecohydrology, hydrochemistry, restoration, stable isotopes, water quality

## Abstract

The reintroduction of beavers in Germany and across Europe has revived interest in their function as ecosystem engineers and their associated impacts on landscapes, hydrological regimes, and water quality. Despite mounting evidence of effects on the hydrology and water quality of rivers, beaver distribution and the links to landscape heterogeneity are still poorly understood. In this study, an extensive intercomparison of two topographically contrasting lowland agricultural catchments was conducted—the Sophienfliess (SOFL) and the Demnitzer Mill Creek (DMC), both in Eastern Brandenburg, Germany. The goal of this study was to assess how catchment structure and functioning mediate beaver activity in terms of distribution and dam density, and how this in turn affects water and nutrient fluxes. A survey of beaver dams in terms of dam location, density, and attributes in both catchments revealed significant differences in the evolution of beaver habitat over time, the number of dams present, and their effects on water flow and nutrient cycling. Most notably, in the lower SOFL catchment, dam density has increased steadily over the last decade and was almost 10 times higher compared to the DMC catchment. In contrast, the recent multi‐year drought in Eastern Germany, as well as differential catchment functioning, has led to reduced beaver activity in the DMC catchment, despite recent wetland rehabilitation efforts. Furthermore, physical differences in dam structure and size could be linked to landscape settings (i.e., valley gradient), surface‐groundwater connectivity, and channel characteristics (straightened vs. meandering). In SOFL, a beaver dam cascade system in the lower catchment propagated significant denitrification, reduction in dissolved organic carbon, and phosphorus concentrations. In contrast, the spatial dynamics of nutrient fluxes in the DMC revealed more localized mobilization processes and strong responses of solutes to hydrological connectivity, which could not be solely linked to beaver activity. Overall, the study showed that catchment structure, hydrological conditions, and stream channel characteristics influence how and where beaver dams are established, and how they in turn influence the physical, chemical, and biological characteristics of surface waters.

## INTRODUCTION

Increasing hydroclimate extremes have intensified concerns over water security in lowland regions across Western and Central Europe, which are vital for agricultural production (Luo et al., 2024; Tetzlaff et al., 2025). Under changing hydroclimate conditions, maintaining agricultural productivity while safeguarding ecological integrity is a central challenge for policy and resource management (Bieroza et al., 2024). Agricultural headwater catchments in particular face persistent pressures from overexploitation of local aquifers, land drainage, changing stream flow regimes, and deteriorating water quality linked to fertilizer inputs (Bieroza et al., 2024; Ying et al., 2025). Considering the projected increased likelihood and severity of extreme floods and droughts, there is a need to develop more sustainable land and water management practices that enhance climate resilience and natural ecosystem functioning (Gutzler et al., 2015; Luo et al., 2024; Smith et al., 2021).

Across much of Europe, decades of extensive land drainage and loss of riparian wetlands due to agricultural intensification have reduced geomorphic and ecological complexity, leaving many lowland systems unable to buffer hydrological extremes and nutrient pollution. This has increased concerns over reduced surface and groundwater storage, increased drought sensitivity, and uncertainty over long‐term water security and agricultural water demands, calling into question past and current land management practices (Ihinegbu & Ogunwumi, 2022; Luo et al., 2024). In drought‐sensitive regions such as eastern Germany, concerns over declining stream flow trends, reduced hydrological connectivity, reduced groundwater recharge, and ecological degradation of lowland ecosystems have renewed interest in sustainable ecosystem management, including ecosystem restoration using nature‐based solutions such as wetland rehabilitation and rewilding practices (Bertassello et al., 2025; Law et al., 2017; Smith et al., 2020; Thompson et al., 2021).

Recognition that degraded freshwater systems can be—at least partially—restored through natural ecosystem engineering has coincided with the reintroduction and recolonization of beavers (*Castor fiber*) in central Europe (Halley et al., 2021). In recent decades, beavers have spread throughout Europe and North America, emerging as an effective restoration agent, capable of restoring geomorphic and hydrological complexity to degraded ecosystems. Through dam building, tree felling, and channel modifications, beavers increase water storage, create wetland habitats, and enhance nutrient retention (Brazier et al., 2021; Fairfax & Westbrook, 2024; Grudzinski et al., 2022; Wohl, 2019). In Germany, beaver reintroductions have been successful, with the Elbe beaver (*Castor fiber albicus*) spreading across the Elbe River system throughout the states of Saxony, Saxony‐Anhalt, and Brandenburg (Halley et al., 2021). In Brandenburg, the current population is estimated between 1000 and 5000 individuals (Hohm et al., 2024). During the last decade, widespread wetland rehabilitation throughout agricultural catchments has further accelerated the autonomous return of beaver (Smith et al., 2020). Despite this, lowland agricultural catchments, impacted by significant alterations to stream morphology and water quality, are not necessarily habitats conducive to beaver settlement. In intensively managed environments, reintroduction and re‐establishment of beavers often generate increased beaver‐human conflicts, while opening up questions about habitat suitability, restoration goals, and timelines, as well as impacts on infrastructure and land use (Auster et al., 2020; Graham et al., 2020; Hohm et al., 2024).

Although the hydrological and ecological benefits of beaver dams are increasingly recognized in upland or forested landscapes (Brazier et al., 2021; Fairfax & Whittle, 2020; Wohl, 2013, 2019), evidence from lowland agricultural catchments remains much more limited. Existing studies have primarily focused on localized effects of individual ponds or immediate downstream impacts (Puttock et al., 2018; Stevenson et al., 2022) or influences within beaver‐impacted wetlands (Fairfax & Westbrook, 2024; Puttock et al., 2018; Smith et al., 2020). Fewer studies have investigated the cumulative, longitudinal impacts of dam cascades across entire catchments, where beaver densities are high (Neumayer et al., 2020). Despite the plethora of beaver‐related studies, there are still knowledge gaps related to the hydrological impacts of beaver dam sequences across different land uses and different hydrological flow conditions (Brazier et al., 2021; Larsen et al., 2021).

That said, there are crucial knowledge gaps regarding landscape controls on beaver distribution and how these may constrain beavers' effectiveness in stream restoration, as not all landscape settings may support high levels of beaver dam activity (Macfarlane et al., 2017). Understanding such interrelationships is crucial for predicting where beavers can sustainably settle, how their engineering modifies catchment hydrology, and assessing whether they can restore ecosystem function in degraded lowland agricultural systems. Previous studies have shown that the size and structure of dams indeed depend on physical factors such as hydrological regime, topography, channel structure, and availability of building materials (Graham et al., 2020; Ronnquist & Westbrook, 2021). However, once a beaver dam is established, it is a relatively persistent structure that remains evident for long periods of time, with long‐lasting effects on the landscape's water balance, ecosystem structure, and biodiversity (Fairfax & Whittle, 2020; Larsen et al., 2021; Moravek et al., 2025; Norman et al., 2022; Puttock et al., 2021; Wohl, 2019). A further research gap also pertains to linking beaver‐mediated hydrological modifications (i.e., flow attenuation, groundwater recharge) with biogeochemical processes (i.e., nutrient retention, carbon storage) at the catchment scale, especially under combined pressures of hydroclimate change and land‐use intensification.

Assessing the effects of the return of beavers to lowland agricultural systems provides the opportunity to generate insights into how and where spatially distributed fertilizer‐enriched runoff, local channel dredging, and disruptive management practices can be mediated by beaver dams and how the effects of such modifications evolve at the landscape scale. The attenuation of flow between beaver dams is one of the key mechanisms that contributes to increased water storage and lateral hydrologic connectivity, affecting hydrological and nutrient fluxes along the entire stream gradient (Brazier et al., 2021). In the context of landscape‐scale impacts of beavers, understanding cumulative downstream effects of dam sequences is therefore essential for guiding ecosystem restoration and beaver reintroductions.

Understanding where dams are likely to be constructed is a challenging but vital task for environmental managers, for maximizing benefits for ecology and hydrology while limiting conflicts and damage to infrastructure (Macfarlane et al., 2017). In this context, this study presents an integrated assessment at the intersection between catchment structure, hydrological processes, and water quality to evaluate the influence of the hydro‐geomorphic landscape context on beaver dam distribution and associated effects on nutrient and dissolved organic carbon (DOC) fluxes in two contrasting agricultural catchments. To this end, we conducted monitoring of water and nutrient concentrations in two agricultural headwater catchments in Brandenburg, in NE Germany—the Sophienfliess (SOFL) and the Demnitzer Mill Creek (DMC) catchments. Both are located in a drought‐sensitive region of NE Germany and characterized by different topography, land‐use distribution, beaver activity, and catchment functioning. The objectives of this study were therefore to (1) investigate how catchment structure, including topography, land use, and streamflow dynamics, influences beaver activity across the stream gradient in each catchment, and (2) assess how water quality and nutrient fluxes are affected across spatiotemporal scales as a result.

## MATERIALS AND METHODS

### Study sites

This study was conducted in two lowland agricultural catchments with contrasting topography and hydrological characteristics in Brandenburg, NE Germany: SOFL and DMC (Figure 1), located ~25 km apart. The region receives ⁓570 mm/year of annual precipitation (1990–2018 long‐term average, DWD, 2023) and an annual potential evapotranspiration (PET) of ⁓650–700 mm/year. Summers are warm (mean 19°C), with convective storms and sporadic high‐intensity rainfall events (>40 mm/day), while winters are cold (mean 0.2°C) with low‐intensity winter rains and <10% of annual precipitation occurring as snow. Since 2013, prolonged below‐average rainfall and a major drought between 2018 and 2022 resulted in declining groundwater levels and increased stream intermittency (Luo et al., 2024; Wu et al., 2022). Agriculture remains the dominant land use in the state (~43%), with fragmented forested areas (primarily Scots pine [*Pinus sylvestris*]) and natural mixed forests and wetlands surrounded by arable land and small urban areas.

**FIGURE 1:**
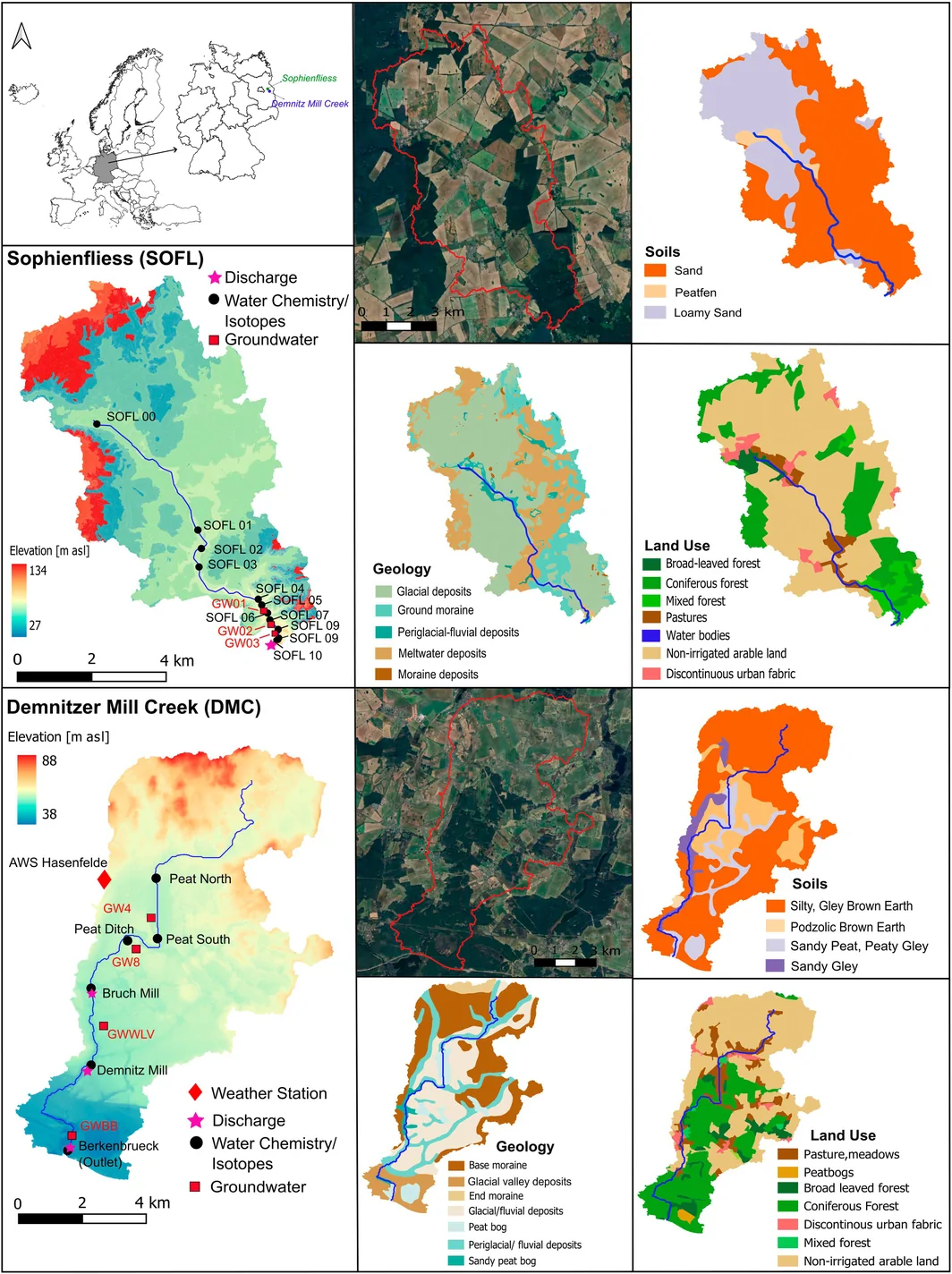
Map of the Sophienfliess and Demnitzer Mill Creek catchment, including geology, soils and land use for each catchment. asl, above sea level; AWS, automated weather station.

#### Sophienfliess

The SOFL catchment (46.9 km^2^) is located in eastern Brandenburg within a protected area (Naturpark Märkische Schweiz), which covers approximately 205 km^2^. The main stream (~10 km long, mean slope of 6.4%) originates from Lake Schlosssee in the village of Proetzel and transitions from an agricultural drainage channel in the upper catchment to a more natural sandy‐bedded lowland river. The lower catchment area is characterized by steep forested hillslopes with postglacial erosion channels and multiple near‐surface groundwater springs. The perennial stream discharges into Lake Schermuetzelsee in the village of Buckow (Figure 1), with a mean discharge of ⁓0.17 L s^−1^ km^2^ (2010–2024 average).

Annual runoff estimates are very low (⁓15 mm/year), which may reflect overestimation of the catchment area based on topography and/or potential losses to deeper groundwater seepage. Regardless, PET in the area frequently accounts for 80%–90% of annual precipitation. Geology in the lower catchment is characterized by peri‐glacial/fluvial deposits, which converge with meltwater deposits, resulting in a number of groundwater springs that emerge in the lower reaches of the stream. Soils are predominantly sandy, leading to high permeability, especially in the mid‐ and lower catchment areas. Land use in the catchment is dominated by agriculture, including arable land and pasture (⁓69.5%), forested areas (⁓27.2%, mixed, coniferous, and deciduous), and ~3% urban fabric. Mean catchment characteristics are summarized in Table 1.

**TABLE 1:** Summary of catchment characteristics, including catchment area (in square kilometers), mean slope (in percentage), total stream length (in kilometers), land use (forest, urban, arable in percentage), and number of observed beaver dams (as of 2025).

Catchment	Catchment size (km^2^)	Slope (%)	Stream length (km)	Land use (%)			No. of beaver dams^c^
				Forest^a^	Urban	Agriculture^b^	
SOFL	46.88	6.4	10.14	27.2	3.3	69.5	35
DMC	66.0	2.0	21.1	36	2.0	60	<10

Abbreviations: DMC, Demnitzer Mill Creek; SOFL, Sophienfliess.

^a^ Forest includes mixed, broadleaf, and coniferous.

^b^ Agriculture includes non‐irrigated arable land and pasture or agricultural grassland.

^c^ Number of beaver dams based on 2024/2025 surveys.

#### Demnitzer Mill Creek

The DMC catchment (68 km^2^), ~25 km south of SOFL, functions as a tributary of the Spree River and serves as a long‐term environmental observatory (Tetzlaff et al., 2023). The entire channel network totals ⁓88 km with a very flat topography (average slope ⁓2%), with the main stream channel accounting for ⁓60 km. The main stream originates as an agricultural drainage channel rather than from a natural lake, like SOFL. Land use is largely dominated by agriculture (⁓60%: nonirrigated crops or grassland), with more forested areas, including conifer monocultures and broadleaf and mixed natural forest in the middle and lower catchment (⁓36%). Extensive riparian rehabilitation occurred in 2000 in the upper catchment (Smith et al., 2020). As a glacially formed lowland landscape, glacial deposits, including moraine material, are present throughout the catchment (Figure 1). Glacial depressions and areas with low relief appear as numerous kettle hole lakes and wetlands. The river network follows fluvial/periglacial deposits, with intermittent riparian peat fens in the mid and lower parts of the catchment. Glacial valley deposits in the downstream area near the outlet at Berkenbrueck (BB) converge with the regional alluvial aquifer system of the Spree River, resulting in strong seasonal surface‐groundwater connectivity.

The stream network is fringed with sandy soils as well as both poorly drained peat and gley soils in the riparian areas. Brown podzolic soils are present in the lowland regions of the catchment, coinciding with a shift towards forest and pasture land. Streamflow in the DMC is groundwater‐dominated and strongly seasonal, with winter maxima and intermittent summer flows. Discharge declines downstream due to groundwater leakage into the freely draining glacial valley deposits. Long‐term discharge at Demnitz Mill (DM) is ~0.1 L s^−1^ km^2^ (2019–2024), while at the outlet, average discharge is 0.01 L s^−1^ km^2^. Annual runoff is very low (<10% of total annual precipitation, <100 mm), due to high annual evapotranspiration (650–700 mm).

### Current beaver activity

After the near extinction of beavers at the beginning of the 20th century throughout Europe, only a few isolated populations remained. One of these remaining populations was in Germany along the Elbe River, with an estimated number of around 200 individuals (Halley & Rosell, 2002). From this isolated population, the first unsuccessful beaver reintroductions to other parts of Germany occurred in the 1930s. In the state of Brandenburg, more widespread and successful reintroductions then followed in the 1960s (Dolch et al., 2002; Hohm et al., 2024). Current numbers for the Elbe beaver (*C. fiber albicus*) in Brandenburg are estimated to be between 1000 and 5000 individuals (Hohm et al., 2024).

In SOFL, beavers were first reported in 2005 near Lake Schlosssee in Prötzel (see Figure 2a). At that time, around 24 active territories were recorded throughout the surrounding nature park, an area of roughly 200 km^2^ (Adlung et al., 2017). Successive mapping of beaver territories has shown a consistent increase in the density and spatial extent of beaver activity, resulting in 64 active territories by 2016. We conducted walk‐over surveys in SOFL in April 2024 and March 2025 to assess the number and state of dams, dominant material, size, and dam structure along the main stream. We noticed several structures in the upper catchment in 2024 were not present in 2025, while new structures were also observed in 2025 (Figure 2a). It is not clear to what extent and by whom beaver dams were potentially removed. Overall, we recorded a total number of 32 structures in 2024 and 38 (including dams and lodges) in 2025. The highest dam density was observed in the postglacial erosion channels of the lower forested portion of the catchment, where ⁓10 active dams/km were observed. The largest dam was observed in the lower catchment with a width of ⁓20 m and an estimated height of ⁓2 m. Other dams were estimated between 2–4 m in width and 0.5–1.5 m in height. Dams were primarily wood‐based, showing signs of ongoing maintenance and high structural integrity, with water flowing through open gaps in the wood construction (see example in Figure 2) or through the base of the dam (Woo & Waddington, 1990). The largest ponded area was estimated to be between 600 and 700 m^2^, with a likely larger inundation extent during high flow periods.

**FIGURE 2:**
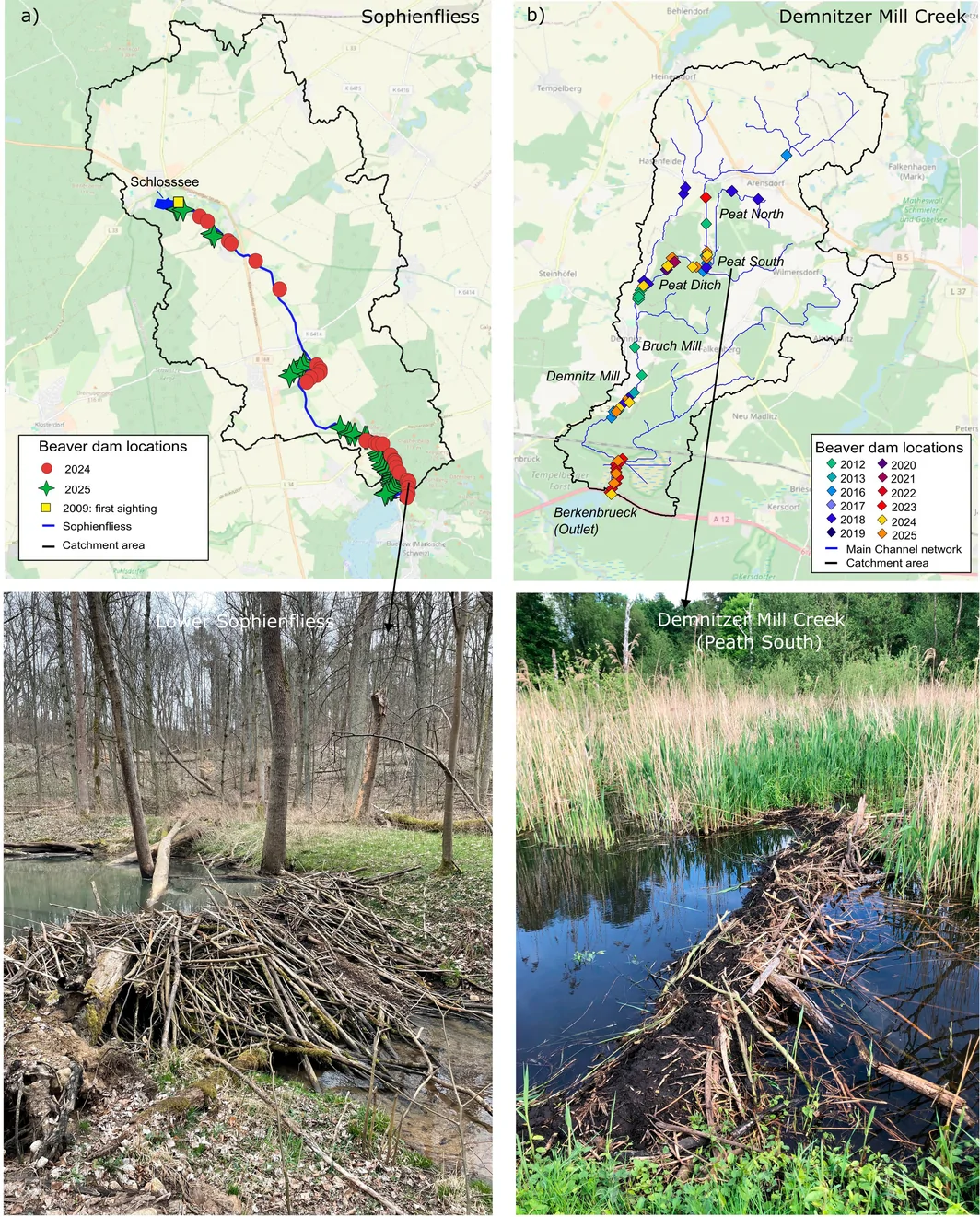
Map of temporal and spatial evolution of beaver activity in the (a) Sophienfliess (SOFL) and (b) Demnitzer Mill Creek (DMC) catchment with typical wood (in lower SOFL, lower left) and mud‐based (Peat South in DMC, lower right) dam structures of each catchment pictured below. DMC map adapted from Grof (2024). Photo credits: Maria Magdalena Warter.

In DMC, beaver activity has been recorded since 2012 by the water and landscape conservation agency Untere Spree (WLV Untere Spree) as well as by Kleine et al. (2021) and Smith et al. (2020). The most recent walkover surveys were conducted by Grof (2024) and during regular sampling of water chemistry. Their presence in the catchment was largely facilitated by the rehabilitation of the wetland area in the upper catchment (Smith et al., 2020) (Figure 2b). Since then, beavers have been present, moving upstream from the catchment outlet at the Spree River into the central wetland area in less than 1 year, and settling in the deeper incised channels and sidearms. Following the 2018–2022 drought, the spatial distribution of beaver activity has been significantly reduced (Grof, 2024, see Figure 2b), with beaver activity largely constrained to three “hotspots”: the restored wetland and riparian area in the upper catchment, as well as riparian areas in the middle and lower catchment (Wang, Tetzlaff, Birkel, et al., 2025). These areas contain a small cluster of primary and secondary structures ranging from one to three dams of varying size and height (see example in Figure 2). Dam density is substantially lower in the DMC, with only 1–3 dams/km and dams being much smaller (max. 1–1.5 m high; 1–3 m wide). The largest dam complex (⁓8–10 m length) was observed in the lower catchment near DM, creating a large inundated wetland area upstream of the dam (~15 ha). In the upstream wetland area (Peat Ditch [PD]), the presence of artificial but abandoned fishponds and relatively undisturbed forest has also resulted in several dam structures and extensive inundation (Wang, Tetzlaff, Birkel, et al., 2025). Dams were predominantly wood‐ and sediment/mud‐based, especially in the wetland areas, where primarily overflow mud‐based structures were observed. In the lower catchment area, near DM, structures were again larger and more wood‐based (Woo & Waddington, 1990).

### Climate and discharge

Daily precipitation is measured by the German Weather Service (DWD) in Muencheberg (Station ID: 3376). In DMC, an automated weather station (AWS Hasenfelde, see Figure 1) recorded precipitation at a 15‐min resolution since 2018 (Tetzlaff et al., 2023). In SOFL, high‐resolution discharge (15 min) was obtained from the LfU for the period 2009 to February 2025. In DMC, discharge has been continuously monitored at three locations: in the wetland (Peat North [PN]), downstream of the wetland in the agricultural area (Bruch Mill [BM]), and downstream of the agricultural area at DM (see Figure 1), with data available from 2019 to 2025, albeit with some data gaps due to sensor issues. At the outlet in BB, daily data were obtained from LfU for the period 2019 until February 2025. At all sites, discharge is measured using pressure transducers at a 15 min resolution, which record water levels (in centimeters). Water levels were translated to discharge (in cubic meters per second) using previously established rating curves (Smith et al., 2020). Daily flow duration curves (FDCs) were developed using the multi‐year data for all sampling stations in each catchment, using daily specific discharge (in liters per second per square kilometer) to assess changes in the flow regime. Flow velocities were determined in the DMC in 2023 (biweekly, January–April) and in SOFL in 2024/2025 (monthly, June–March), using an impeller device (OTT MF Pro water flow meter, OTT Hydromet), measuring velocities at two heights across stream cross sections. In the DMC, velocities were measured at PN, Peat South (PS), and DM, while at SOFL, velocities were measured at SOFL10 near the outlet.

### Water quality, hydrochemistry, and stable water isotopes

In SOFL, collection of stream water samples started on a biweekly basis in February 2024, shifting to monthly sampling from April 2024 to February 2025. A total of 11 sites were sampled, nested along the stream network in SOFL, with the selection of sites based on spatial coverage and accessibility (see Figure 1). Synoptic sampling of groundwater springs and tributaries for isotopes and water quality was carried out in March and November 2024, as well as in February 2025. In the DMC, a total of nine nested sampling sites have been well established since 2017, which are routinely sampled on a weekly basis. In this study, we primarily focused on six sites, including PN, PS, PD, BM, DM, and BB. In each catchment, grab samples were collected on a weekly basis for analysis of physicochemical water quality indicators (e.g., temperature, conductivity, pH, dissolved oxygen [DO]) and hydrochemistry, as well as stable water isotope ratios of oxygen and hydrogen (δ^18^O and δ^2^H). Basic water quality parameters (pH, water temperature, DO, and electrical conductivity [EC]) were measured using a handheld multiparameter probe (WTW Multi 3630 IDS; Xylem Analytics, Weilheim, Germany). See Appendix S1: Table S1 for a detailed overview of sampling periods.

For stable isotope analysis, grab samples from the stream were filtered (0.22 μm cellulose acetate) into 1.5‐mL vials (LLG Labware) and analyzed using Cavity Ring‐Down Spectroscopy with a Picarro L2130i Isotopic Water Analyzer (Picarro Inc., Santa Clara, CA). We calculated line‐conditioned excess (lc‐excess), which defines the deviation of the relationship between δ^2^H and δ^18^O from that of precipitation, indicating evaporation impacts (Landwehr & Coplen, 2006). In addition, we used precipitation isotopes from the AWS in Hasenfelde in DMC and stream water isotopes from each catchment to estimate damping ratios (DRs) at each sampling location. As a ratio of the CV of δ^18^O or δ^2^H in streamflow to that of δ^18^O or δ^2^H in precipitation, DRs can be used as a semiquantitative proxy measure to assess general trends and distributions of transit times within and across multiple catchments (Tetzlaff et al., 2009). For reference, a lower DR generally indicates longer transit time dynamics, while higher DRs are indicative of a fast‐responding system.

Hydrochemistry analyses focused on nutrient‐based inputs of nitrogen and phosphorus in the form of nitrate‐nitrogen (NO_3_‐N), soluble reactive phosphorus (SRP), as well as DOC and sulfate (SO_4_). SRP, together with NO_3_‐N, was analyzed from filtered and acidified (2 M HCl) samples by flow segmented analysis. DOC was measured in filtered samples by infrared spectrometry after high‐temperature oxidation (Shimadzu TOC‐V and ‐L, precision ⁓3%), while sulfate concentrations were determined through ion chromatography after chemical suppression. Normalized chemical fluxes (*F*) were calculated based on concentrations (*C* in micrograms per liter), concurrent discharge (*Q* in cubic meters per second), and sub‐catchment area (*A* in square meters) for each catchment as:

F=C×Q/A.

The main results present catchment‐normalized nutrient and DOC fluxes (in kilograms per day per square meter) for the period of January 2024–February 2025, as this was the time period that concurrent discharge data were available in each catchment. Raw concentrations (in micrograms per liter) data for the period January 2024–December 2025 can be found in Appendix S1: Figures S3 and S4.

### Statistical analyses

Data analysis was performed in Python (v. 3.13.7) and R (v. 4.4.3). Baseflow separation was done using the open‐source *hydroEvents* package in R, which uses the one‐parameter recursive digital filter approach by Lyne and Hollick (1979) to separate streamflow into baseflow and quickflow (Wasko & Guo, 2022). Linear mixed‐effects models (*lmer* function in R) were used to assess the influence of catchment characteristics (i.e., slope, beaver dam density, land use, groundwater influence) on within‐catchment differences in DO dynamics and SRP, DOC, NO_3_‐N, and SO_4_ concentrations across the stream gradients. We used mixed models with seasonality and sample locations as random effects to account for repeated monthly sampling and potential seasonal variability at each site. Fixed effects (predictors) included catchment characteristics such as slope, land use, and dam density, as well as water quality parameters such as pH, water temperature, and Ca concentrations, which served as proxies for hydrological connectivity (i.e., groundwater inflow). Land use was included as a discrete variable (i.e., forest, arable, wetland) while other parameters were treated as numerical vectors. Numerical vectors were log‐transformed to account for differences in measuring units. The significance of continuous predictors (i.e., slope, beaver dam density) was evaluated using mixed‐effects regression coefficients and likelihood ratio tests (*anova*). Categorical predictors (i.e., land use) were assessed through post hoc pairwise comparisons among land‐use categories using Tukey‐adjusted estimated marginal means (*emmeans*). We used raw concentration data for mixed‐effects modeling. Beta coefficients and their significance level are reported for each parameter included in the final model.

## RESULTS

### Rainfall‐runoff responses and flow regimes

Following the 2018–2023 drought, 2024 was an overall wet year in Germany. However, some regions—such as Berlin and Brandenburg—still experienced a precipitation deficit, despite a wet winter and rain‐intensive summer. Total annual precipitation in 2024 for the state of Brandenburg was 601 mm (DWD, 2025), with regional differences. Total annual precipitation in 2024 was 630 mm and 570 mm in DMC and SOFL, respectively. The subsequent 2024/2025 winter and 2025 spring were again much drier, with total annual precipitation of 496 and 463 mm in DMC and SOFL, respectively. FDCs of long‐term discharge (2019–2024) in SOFL indicate a perennial streamflow regime with limited flashiness (Figure 3a) and higher stream velocities (Figure 3b) during high flows. Mean long‐term specific discharge was ~0.1 L s^−1^ km^2^ (2019–2024 average) (Figure 4a).

**FIGURE 3:**
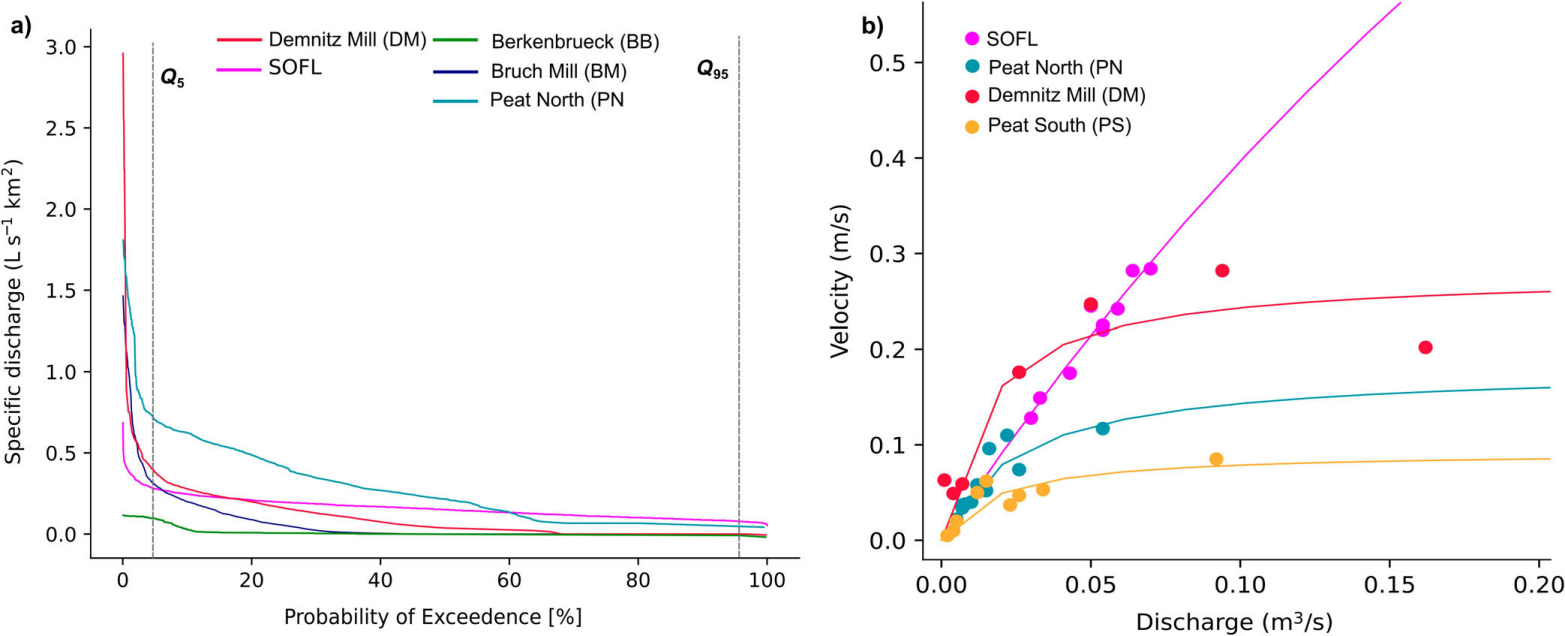
(a) Flow duration curves (FDCs) of the Sophienfliess (SOFL) and Demnitzer Mill Creek (DMC) catchments using daily streamflow data. (b) Velocity measurements from stage‐discharge measurements in the SOFL and selected sites in DMC.

**FIGURE 4:**
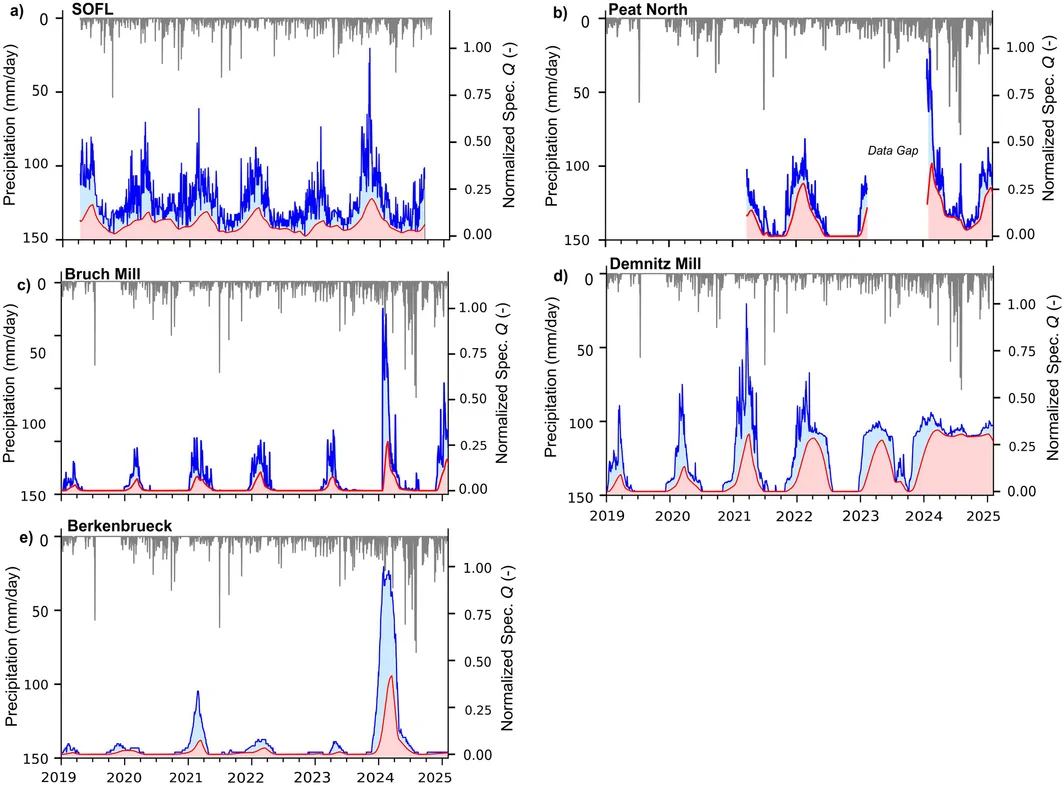
Time series of daily precipitation (gray) and normalized discharge (blue) for sites in the (a) Sophienfliess (SOFL) and (b–e) Demnitzer Mill Creek (DMC): (a) lower SOFL, (b) Peat North, (c) Bruch Mill, (d) Demnitz Mill, and (e) Berkenbrueck. Baseflow is indicated in red, stormflow is indicated in blue. Data at DMC24 are only available from 2021, and a data gap between 2023 and 2024 is indicated.

Overall, low flow periods occurred primarily in summer and autumn, while flow maxima were observed in winter. The wet winter in early 2024 was reflected in increased winter discharge (up to 0.4 L s^−1^ km^2^), with flows declining subsequently throughout spring and summer. At around 8 km, a hydrological transition zone appears, with sections of the stream drying out in June and July 2024, despite high rainfall, but consistent with regional high summer evapotranspiration and soil moisture deficits. A high mean baseflow index (BFI; mean 0.68, min = 0.29, max = 0.9, 2019–2024 average) indicates a strong baseflow component driven by local groundwater contributions. Runoff was estimated to be only around 3% of annual precipitation.

In DMC, streamflow is characterized by distinct spatial variability and strong seasonal streamflow responses, with winter maxima and cessation of flows for most years in summer (Figure 3a). High flows typically correspond to high precipitation inputs and are largely driven by transient, rapid near‐surface runoff from drained agricultural soils. A steeper FDC slope in PN indicates higher responsiveness of flows, with low flow periods more frequent in the lower catchment, where hydrologic connectivity is much more seasonal (Figure 3a). Flow velocities are overall slower (mean 0.08 m/s) due to the flatter topography (Figure 3b). At BM and DM sites, streamflow was highly seasonal and strongly coupled to seasonal surface‐groundwater connectivity (Figure 4c,d). The comparatively wet conditions of the 2024/2025 winter resulted in increased streamflow throughout the entire DMC catchment and overall perennial flow conditions (Figure 4b–d). This was a marked contrast to previous drought years, where flow cessation typically occurred between April and October. At all sites, baseflows were highly variable, ranging between 0.3 and 0.7 with distinct seasonal differences, highlighting the spatiotemporal variability in groundwater contributions. Overall, runoff was ~10% of annual precipitation, owing to the high evaporative loss rate (which may average ~80%) in the region. Mean multi‐year discharge statistics for both catchments for the 2019–2024 period are summarized in Table 2.

**TABLE 2:** Summarized mean discharge statistics (2019–2024) for the outlet in Sophienfliess (SOFL) (SOFL10) and multiple sites in Demnitzer Mill Creek (DMC), including mean daily specific discharge (*Q*_mean_), maximum and minimum discharge (*Q*_max_, *Q*_min_) (all in liters per second per square kilometer), 5th and 95th discharge percentiles (*Q*_5_ and *Q*_95_), mean annual runoff (*Q*_RO_ in millimeters), annual mean baseflow index (BFI), and annual mean runoff coefficient (RC as percentage of annual precipitation [P]) for the study period.

Location	*Q*_mean_ (L s^−1^ km^2^)	*Q*_max_ (L s^−1^ km^2^)	*Q*_min_ (L s^−1^ km^2^)	*Q*_5_ (L s^−1^ km^2^)	*Q*_95_ (L s^−1^ km^2^)	*Q*_RO_ (mm/year)	BFI (‐)	RC (%)
SOFL10	0.13	0.28	0.04	0.16	0.06	11.2	0.68	3
Peat North	0.35	1.8	0.06	0.8	0.06	27.8	0.74	6
Bruch Mill	0.07	1.46	0	0.34	0	14.8	0.32	5
Demnitz Mill	0.1	0.55	0	0.21	<0.01	14.5	0.48	3
Berkenbrueck	0.01	0.11	0	0.04	0	1.7	0.27	<1

### Catchment stable water isotopes

Precipitation isotopes from the AWS Hasenfelde were assumed to be broadly representative of both catchments, showing characteristic seasonal variability: depleted winter signatures (δ^18^O = −10.3; δ^2^H = −72.2) and enriched summer values (δ^18^O = −5.1; δ^2^H = −33.5) (Figure 5a). Spatiotemporal patterns of isotopic signatures of stream water reveal heterogeneity in contributing water sources (i.e., groundwater, tributary streams, surface inflows) and stream‐groundwater connectivity between the upstream agricultural and beaver‐impacted downstream reaches.

**FIGURE 5:**
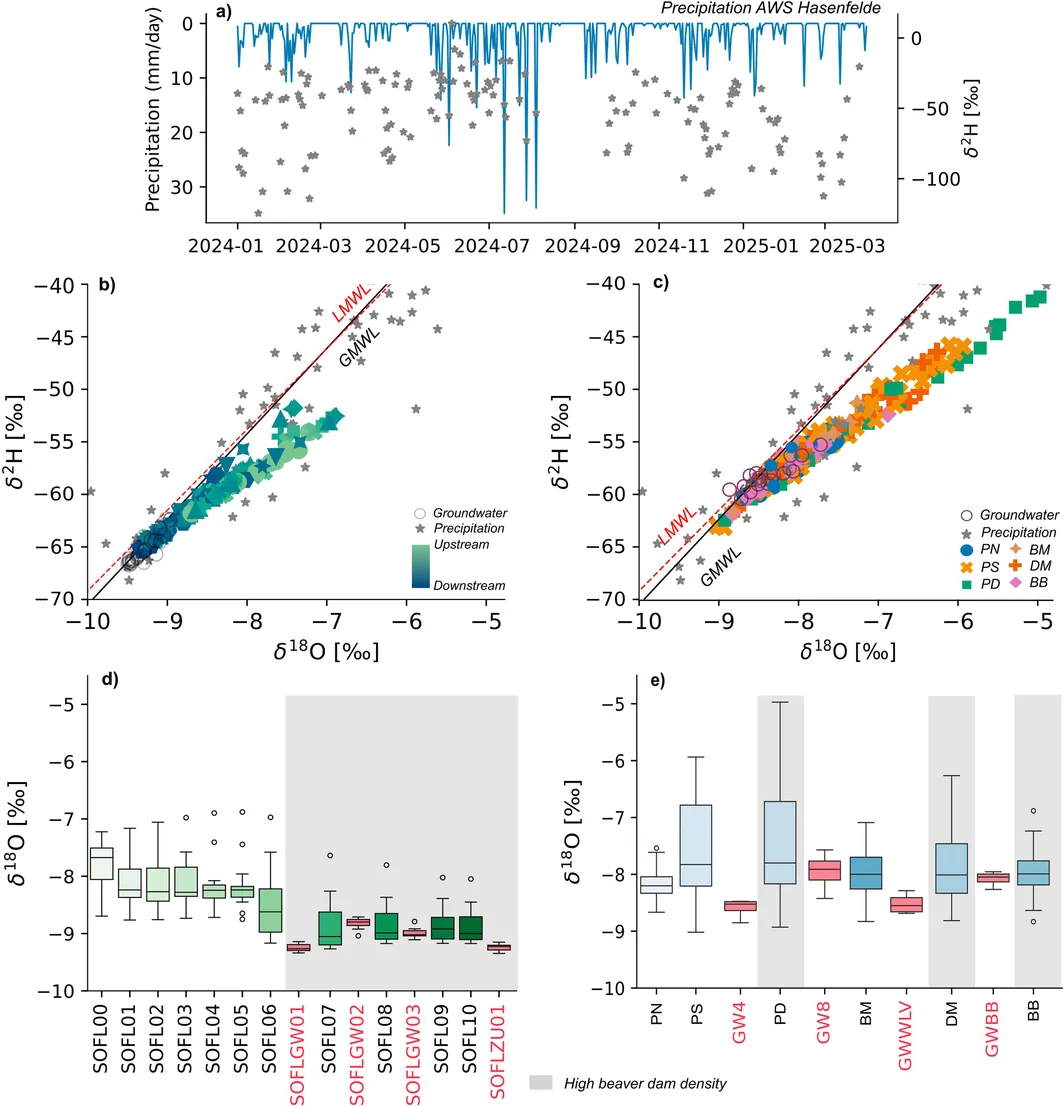
(a) Daily precipitation and precipitation isotopes from the automated weather station (AWS) Hasenfelde (2019–2026), which is broadly representative of both catchments. (b) and (c) Dual isotope plots of Sophienfliess (SOFL) and Demnitzer Mill Creek (DMC). In SOFL, upstream to downstream sites are sorted by color gradient (light to dark, respectively). Sample sites in DMC are colored and highlighted with different symbols. Global (black) and local (red) meteoric water lines are indicated. (d) and (e) Boxplots of spatial variability of δ^18^O isotopes for the SOFL and DMC across all sample points, including local groundwater sample sites (in red). Areas with high beaver activity are shaded. BB, Berkenbrueck; BM, Bruch Mill; DM, Demnitz Mill; GMWL, Global Meteoric Water Line; LMWL, Local Meteoric Water Line; PD, Peat Ditch; PN, Peat North; PS, Peat South.

In SOFL, stream water isotopes were more damped relative to precipitation, with downstream depletion reflecting increased groundwater and tributary inflows (Figure 5d). The most enriched signatures were observed at the stream inlet in December 2024 (Figure 5b, see also Appendix S1: Figure S1), likely reflecting flushing of fractionated lake water. Similarly, after multiple stream sites in the middle catchment were dry in June and July, enriched upstream water was flushed downstream during a period of brief reconnection in August, before flows ceased again in September and October 2024. Overall, upstream sites were more isotopically enriched, while downstream locations showed consistently more depleted signatures and more damped responses. DRs were ~0.1 upstream and <0.1 downstream, indicating stronger groundwater influence and longer transit times in the lower catchment (see Appendix S1: Table S3).

In DMC, stable isotopes from wetland sites showed strong variability, with progressive enrichment from the PN to PS, where multiple beaver dams are located (Figure 5c,e, see also Figure 2b). Winter signatures largely resembled local groundwater and/or winter precipitation, whereas low‐flow periods showed strong evaporative enrichment. Strongest enrichment was observed in the wetland area, where several old fish ponds are connected to the stream and are now flooded by beaver dams, storing water during low‐flow periods.

Downstream of the wetland at DM, enriched water was flushed from inundated wetland areas, while at BB a geological divide strongly drives greater surface‐groundwater connectivity, resulting in distinctly depleted signatures with limited variability (Figure 5c, see also Appendix S1: Figure S1). DRs in DMC decreased along the network, with DR upstream of the wetland at PN ~0.24, reaching up to ~1.0 at PS, then decreasing again throughout the lower catchment. Near the catchment outlet at BB, DR was ~0.14, confirming the heterogeneity in stream connectivity and longer residence times (see also Appendix S1: Table S3).

### Spatiotemporal dynamics in water quality

In line with stable isotope signatures in the SOFL, distinct spatial differences between the upstream agricultural and downstream beaver‐impacted areas were observed (Figure 6a–d). Overall, pH was alkaline (mean 7.9), with the highest values observed in the hydrological transition zone at ~8 km, while in the lower catchment, pH was less variable. EC showed a downstream increasing trend (mean upstream: 595 μS/cm; mean downstream: 665 μS/cm). DO exhibited a slight increasing trend from the inlet until the transition zone, before stabilizing in the downstream area (mean DO: 10.6 mg/L) (Figure 4c). Synoptic samples of groundwater quality (see also Appendix S1: Figure S2) from springs in the lower SOFL catchment indicated the presence of underlying solute‐rich minerogenic meltwater deposits and peri‐glacial/fluvial deposits, due to increased EC (mean: 670 μS/cm). Stream water temperature was higher upstream (mean 10.3°C) compared to downstream (mean 9.1°C), reflecting the inflow of cooler groundwater (mean 9.4°C) and forest shading.

**FIGURE 6:**
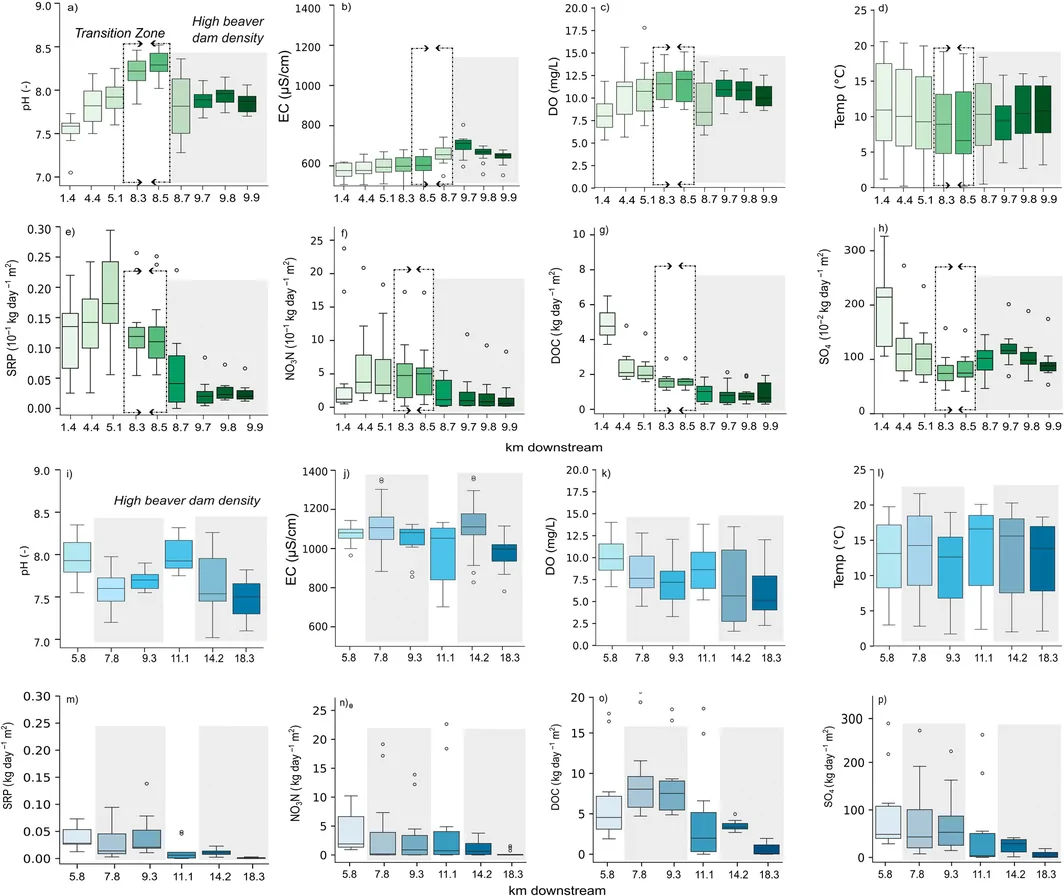
Boxplots of water quality (a–d) and normalized chemical fluxes of soluble reactive phosphorus (SRP), nitrate‐nitrogen (NO_3_‐N), dissolved organic carbon (DOC), and sulfate (SO_4_) (e–h) in the Sophienfliess and Demnitzer Mill Creek catchment (i–l and m–p), respectively. Note the difference in magnitude for SRP (10^−1^), NO_3_‐N (10^−1^), and SO_4_ (10^−2^) for Sophienfliess. Areas of high beaver activity are indicated through gray shading. The hydrological transition zone with intermittent stream flow in the middle SOFL catchment is highlighted between kilometers 8.3 and 8.5. DO, dissolved oxygen; EC, electrical conductivity.

In DMC (Figure 6i–l), pH was alkaline (mean 7.8), but declined throughout the upstream wetland area and the lower catchment. Conductivity was higher in DMC compared to SOFL (mean: 1036 μS/cm), indicating greater ionic strength throughout the catchment. DO levels declined between the upstream wetland area and the catchment outlet (up to 60% reduction). Large within‐site variability in DO levels further indicated, at times, significant seasonal deoxygenation in the lower catchment (min: 1.64 mg/L). Mean water temperature was 13°C (Figure 6k), with no significant differences between sites.

### Seasonal dynamics in nutrient and DOC fluxes

Given the contrasting distribution of beaver dams and spatial hydrologic connectivity in both catchments, distinct spatiotemporal patterns of nutrient and DOC fluxes emerged (see also Appendix S1: Figures S3 and S4 for raw concentrations). In SOFL, NO_3_‐N, SRP, and SO_4_ fluxes exhibited significant upstream‐downstream differences (all *p* < 0.001). Notably, SO_4_ and DOC fluxes were higher at the stream inlet (Figure 6g,h), consistent with a significant source of organic matter input from the minerotrophic peat soils in the surrounding wetland. However, both declined throughout the lower catchment, with DOC decreasing by more than 80% between the inlet (5 kg day^−1^ m^2^) and outlet (0.9 kg day^−1^ m^2^), indicating strong biogeochemical cycling along beaver dam cascades. SO_4_ exhibited slight increases in the lower catchment, while SRP and NO_3_‐N fluxes first increased within the upstream area, with higher concentrations of fertilizer‐based nutrients primarily in winter and spring (~2 × 10^−1^ kg day^−1^ m^2^) and lower concentrations during the main growing period (<1 × 10^−1^ kg day^−1^ m^2^). NO_3_‐N significantly declined throughout the lower catchment by almost 50%, indicating increasing denitrification processes, as well as potentially increasing biological uptake during the growing season throughout the lower catchment. Similarly, the decline of SRP throughout the lower catchment (Figure 6e) indicated significant P retention by either biological uptake or sediment processes within beaver ponds.

In the DMC, fertilizer‐based nutrient levels (i.e., NO_3_‐N) were up to 10 times higher (between 5 and 10 kg day^−1^ m^2^) than in SOFL (0.5–14 kg day^−1^ m^2^). SRP decreased by up to 80% between the wetland area and the catchment outlet. Lowest fluxes were recorded at the outlet (0.0008 kg day^−1^ m^2^), likely due to source limitations in nonagricultural areas further downstream (Figure 6m). SRP export increased in summer in the wetland area (up to 0.14 kg day^−1^ m^2^), peaking during a rain‐intensive period in summer, likely as a result of increased mobilization of phosphates in channel/riparian soils in response to precipitation and increased surface runoff.

Similar to fertilizer‐based inputs, DOC fluxes in the DMC were more enriched in the upstream wetland area, increasing by up to 70% between the upper and lower boundaries of the wetland, and reaching up to 10 kg day^−1^ m^2^ (Figure 6o). The richness of peat soils in the wetland area likely represented a ubiquitous source of DOC; especially under wet conditions in winter and spring, DOC was increasingly mobilized throughout the wetland, reaching export fluxes of up to 20 kg day^−1^ m^2^. In contrast, in the lower catchment, DOC was low all year round with limited variability (mean ⁓4 kg day^−1^ m^2^), suggesting increased metabolic breakdown in the lower stream network where beaver ponds and wetland‐like sections are present. SO_4_ fluxes were up to five times higher in the upper catchment (maximum flux 250 kg day^−1^ m^2^) in winter, but declined rapidly during spring as streamflow declined (Figure 6p, see also Appendix S1: Figure S4). A significant reduction of SO_4_ by ⁓90% could be observed between the upstream wetland (mean 91 kg day^−1^ m^2^) and catchment outlet (mean 5.5 kg day^−1^ m^2^), possibly reflecting increased oxidation of sulfur in drained wetland soils.

### Influence of stream connectivity, topography, and land use on beaver activity

Mixed‐effects modeling of nutrient concentrations revealed solute‐specific controls in both catchments, in which beaver dam density, groundwater influence (indexed by Ca concentrations), and geomorphic context (slope) jointly regulate DO dynamics and nutrient and DOC fluxes (Figure 7). In SOFL, beaver dam density had a strong positive effect on DOC (*p* < 0.001), but a strong negative effect on SRP, NO_3_‐N, and SO_4_. This would indicate that beaver impoundments in the SOFL function as sources of organic carbon due to increased input from surrounding riparian areas, but act as sinks for inorganic nutrients and sulfate. Groundwater inflow had a strong negative effect on DOC (*p* < 0.001) and SRP (*p* < 0.001), a strong positive effect on SO_4_ (*p* < 0.001), but no significant effect on NO_3_‐N. In addition, for SRP, strong negative relationships with hydrologic and geomorphic variables (all *p* < 0.01) suggest that while beaver‐driven retention was strong across the longitudinal stream gradient, the inflow of Ca‐rich groundwater in the lower catchment, as well as seasonal phosphorus cycling and hydrologic variability, were equally dominant controls. Notably, spatial patterns of NO_3_‐N appeared primarily linked to dam density and slope (both *p* < 0.01), while hydrological predictors did not show significant effects. This would suggest that denitrification was mainly driven by the high beaver dam density. In contrast, for DOC, all predictors were significant, indicating that DOC concentrations were driven by a combination of beaver‐mediated removal, hydrologic connectivity, groundwater contributions, and seasonal remobilization. Similarly, DO dynamics in SOFL appeared to be jointly modulated through beaver dams and water source mixing related to turbulent flows across beaver dam cascades.

**FIGURE 7:**
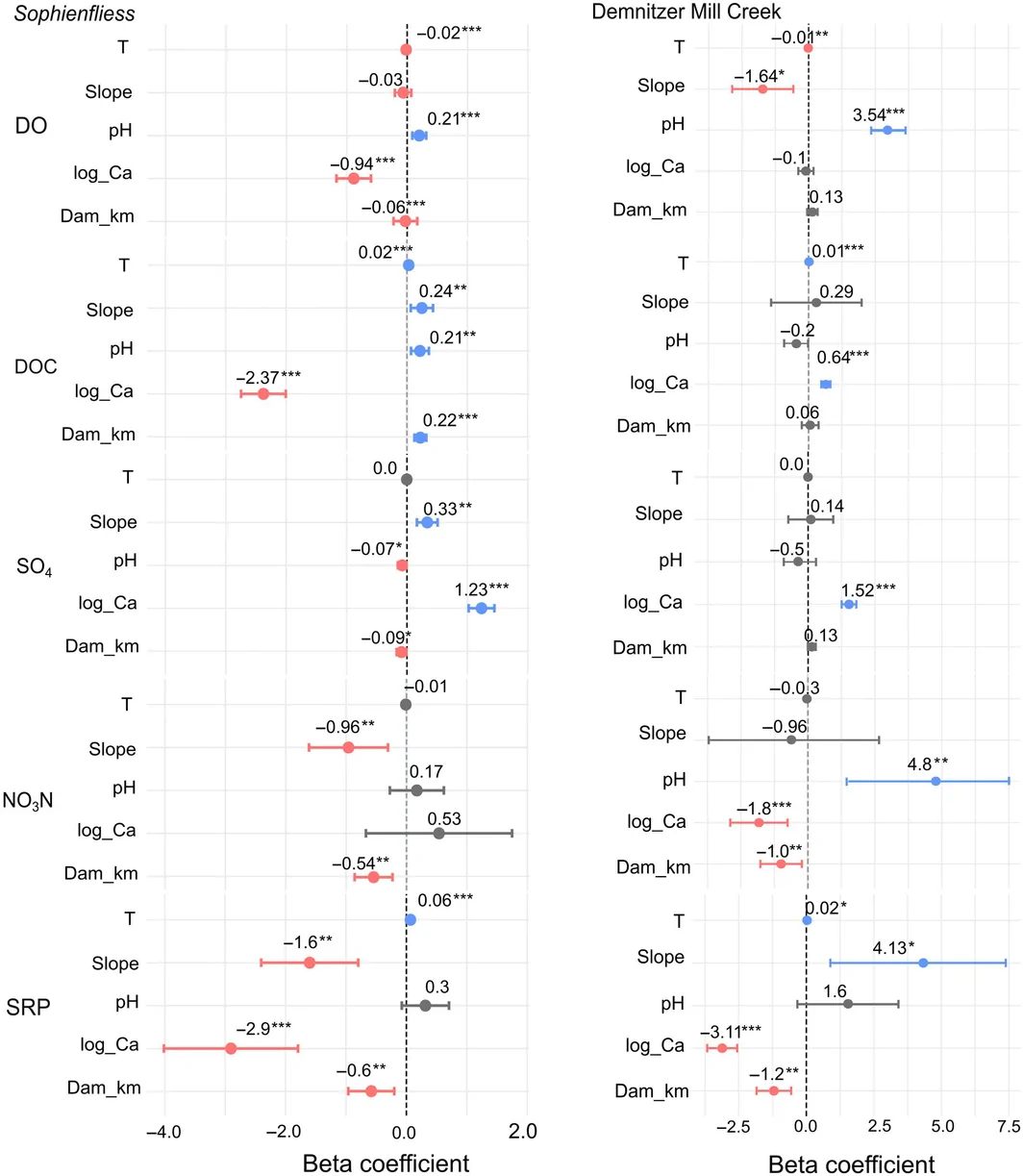
Linear mixed‐effects model beta coefficients of Sophienfliess (left) and Demnitzer Mill Creek (right) and their 95% CIs. The coefficients correspond to the fixed effects in the final linear mixed‐effects models for the effects of landscape structure (slope), beaver activity (dam density), and hydrological connectivity (pH, log_Ca) on dissolved oxygen (DO), dissolved organic carbon (DOC), sulfate (SO_4_), nitrate‐nitrogen (NO_3_‐N), and soluble reactive phosphorus (SRP) dynamics. Colors indicate positive (blue) or negative effect (red), with asterisks denoting significance level at the 0.5 (*), 0.01 (**), and 0.001 (***) level, while not significant parameters are in gray.

In DMC, dam density had a strong negative effect on SRP and NO_3_‐N but only a weak positive effect on SO_4_. This suggests that beaver‐impacted wetland areas were effective phosphorus and N sinks, but not as strong sulfate sinks as in the SOFL. In terms of DOC, neither beaver dam density nor slope had a significant effect in the DMC. However, groundwater influence had a strong positive effect (*p* < 0.001), signifying a stronger coupling with groundwater discharge and DOC mobilization, especially from the organic‐rich wetland and peat soil in the upstream area of DMC. Negative effects of slope and temperature and positive effects of pH on DO dynamics, suggest a stronger influence of seasonal hydrologic conditions rather than beaver activity.

Notably, land use only showed a strong positive effect on DOC in both catchments, suggesting that in‐stream processing and organic and groundwater inputs largely override landscape source signals for other nutrients. In both catchments, water temperature had a positive effect on the seasonal dynamics of DOC (*p* < 0.001) and SRP (*p* < 0.001), but not on NO_3_‐N or SO_4_.

In DMC, groundwater inflow had a strong negative effect on SRP (*p* < 0.001), as well as NO_3_‐N (*p* < 0.001), but a strong positive effect on SO_4_ and DOC (both *p* < 0.001). The combined negative effects of beaver dam density and groundwater on SRP and NO_3_‐N in DMC suggest that both hydrologic (groundwater) and beaver‐mediated processes strongly interact. However, the lack of effect of dam density on DOC and SO_4_ would mean that hydrologic connectivity is more affected by catchment differences in source waters and hydrologic connectivity rather than by biologically beaver‐mediated in‐stream processing (see Appendix S1: Table S4 for model summary).

## DISCUSSION

### Influence of catchment structure and landscape settings on beaver activity

This paired catchment study provided new insights into how catchment structure and functioning shape the distribution of beaver dams and how—in turn—their cumulative longitudinal impacts affect hydrology and nutrient fluxes. From the analysis of physical and chemical parameters, the different catchment structures and functioning of SOFL and DMC catchments—in terms of landscape setting, land use, channel gradients, stream character, and riparian habitats—resulted in contrasting patterns of beaver activity and nutrient and DOC fluxes. While both catchments are dominated by agriculture in their upstream areas and more natural, forested vegetation downstream, differences in geomorphology, hydrological connectivity, and streamflow regimes created contrasting ecohydrological boundary conditions.

Considering the preference of beavers for gentle slopes with perennial slow‐flowing waters and proximal riparian forest (Brazier et al., 2021; Ronnquist & Westbrook, 2021), it is not surprising that beavers were highly concentrated in the lower SOFL catchment. The forested, meandering, low‐gradient stream represents ideal beaver habitat compared to the channelized agricultural stream in the upper catchment. Consequently, the density of beaver dams and the observed structural integrity and height of dams throughout the lower stream system signify that hydrologic and habitat conditions were stable and suitable enough for long‐term settlement of beavers. In addition, perennial flows, maintained by local groundwater springs, further provide the kind of hydrological boundary conditions that represent high‐quality beaver habitat. In turn, this created a feedback loop where existing surface‐subsurface connectivity was likely further enhanced by beaver populations increasing water residence times and retention (Fairfax & Westbrook, 2024; Polvi & Wohl, 2012). As such, while lateral connectivity in the SOFL may be limited by the steeper slope, vertical connectivity between the surface and subsurface is especially high, as evidenced by more depleted isotopic signatures and lower DRs.

In DMC, the dominance of straightened drainage channels throughout the catchment, as well as a highly seasonal streamflow regime and discontinuous riparian areas, would appear less suitable for beaver habitat. Despite this, restoration of the upstream wetland area in the early 2000s, followed by several consecutive wet years, initially accelerated the dispersal of beavers throughout the DMC channel network (Smith et al., 2020). That said, following this initial expansion (see Figure 2b), a drought‐induced decline in stream flows and increased intermittency following the recent 2018–2023 multi‐year drought have led to a significant retreat of beavers to isolated riparian areas with high saturation (Grof, 2024). Despite multiple examples of beavers' ability to revitalize and buffer ecosystems against the negative impacts of climate change (Fairfax & Westbrook, 2024; Hood & Bayley, 2008; Moravek et al., 2025), this drought‐induced retreat would imply that even a highly adaptable species such as the beaver may be constrained by extreme hydrologic stress (Goldfarb, 2018; Wohl, 2019).

Considering the differences in dam morphology, our results also align with previous research on dam structures and composition. The differences in wood‐based versus more sediment‐based dams confirm the adaptation of beavers in dam construction based on the local physical environment and flow conditions (Ronnquist & Westbrook, 2021; Woo & Waddington, 1990). The observed spatial variability of materials (mud, smaller branches) used for dam construction in DMC would reinforce the influence of catchment and landscape settings on dam geometry, with dams in the upstream peat fen being more mud‐ and sediment‐based, while in the downstream riparian area, wood‐based dams were dominant. Such adaptive behavior has been observed across different landscapes, with beavers using anything from rocks to loose debris to construct and reinforce dam structures in place of other materials and according to flow conditions (Fairfax & Westbrook, 2024; Jung & Staniforth, 2010). Also, the reduced dam height and width in the DMC, compared to SOFL, matches the straightened channel structure, very flat topography, and slow flow velocity. Under such conditions, dams are required not to be as high and wide but rather to be more impermeable to support the extension of ponded areas beyond channel borders and maintain water levels. In this way, lateral connectivity is likely higher in DMC, with considerable inundation areas and floodplain connectivity observed in the upstream wetland (Wang, Tetzlaff, Birkel, et al., 2025). In contrast, in the SOFL, the steeper topography, faster flows, and higher channel gradient require more compact, porous structures (i.e., wood‐based) that can withstand higher flow velocities and mediate potential hydraulic stress during high flow periods (see Figure 3b) (Ronnquist & Westbrook, 2021).

### Impact of beaver dams on biogeochemistry and hydrologic connectivity

Even though this study only covers one annual cycle, it is evident that landscape settings, hydrogeology, and hydrological conditions mediated beaver activity and, in turn, nutrient and carbon cycling processes. In lower SOFL, the beaver dam cascades have increased flow attenuation, mediating surface‐groundwater connectivity and increasing residence times, as evidenced by lower DRs and the upwelling of multiple springs in the lower catchment. At the same time, commonly found decreases in nitrogen and phosphorus in beaver‐impacted systems were evident (Grudzinski et al., 2022; Puttock et al., 2017), albeit with differential mechanisms. While denitrification processes appear primarily linked to beaver‐driven retention, hydrological connectivity was influential in DOC and phosphorus retention. Notably, the significant reduction of DOC along the stream gradient in SOFL diverges from many other studies. Arguably, the majority of these studies focus on concentrated wetland and riparian areas, where increased output of DOC downstream of beaver ponds was noted. This has been generally associated with an increase in wetland area, increased primary production in beaver ponds (Larsen et al., 2021; Puttock et al., 2017, 2018), accumulation of organic matter and increased carbon availability from microbial respiration (Wegener et al., 2017), and/or retention of particulate organic carbon and litter decomposition (Law et al., 2016). The decreasing trend of DOC—as observed in SOFL—is less common elsewhere, at least in agricultural catchments. One of the few studies observing this is Kothawala et al. (2006), who linked reduced DOC export along a stream to a headwater wetland acting as a significant source of DOC, which was then subsequently reduced downstream due to beaver ponds. Considering the presence of a wetland area at the source of SOFL, the patterns of increased DOC concentrations at the stream inlet thus correspond to Kothawala et al. (2006). The subsequent source limitation and groundwater inflow in lower SOFL appear to further contribute to DOC dilution, with beaver dams playing a complex dual role in terms of increasing hyporheic exchange and biogeochemical processing. Meanwhile, in DMC, higher concentrations in the upstream and downstream wetlands were more likely driven by enhanced mixing of surface and subsurface water within the upstream wetland, in addition to mobilization of organic matter input from organic‐rich substrates (Wang, Tetzlaff, Birkel, et al., 2025; Wu et al., 2021). It is possible that the shorter biogeochemical record of this study may not fully capture inter‐ and intra‐annual variability and hydroclimate responses, which are key drivers of flow conditions in DMC (Wang, Tetzlaff, Birkel, et al., 2025; Warter et al., 2024). However, it also clearly illustrates that the hydrological and landscape‐specific context are dominant controls in DMC.

Most surprising were the contrasting patterns of consistently higher DO in the beaver‐impacted lower catchment in SOFL, while highly variable and seasonally hypoxic conditions were observed in DMC (see Figure 5). This apparent dichotomy of oxygen concentrations in beaver‐impacted streams implies that the influence of beaver dams on oxygen conditions may not be as ubiquitous as expected and is strongly linked to flow conditions. As such, our prevailing hypothesis is linked to observed differences in physical and hydrological conditions (stagnant vs. turbulent flow), as well as dam structures and flow states. Based on the physical settings in SOFL—the steeper valley gradient, plus higher flow velocity, and larger, more permeable dams—we hypothesize that the more turbulent cascading flowing action across wooden debris from different heights potentially fosters greater aeration of stream water. Analysis of multiple annual cycles of DO patterns and flow variability could give different relationships and insights into in‐stream effects of long‐term beaver activity.

The distinctly different DO patterns in DMC led us to hypothesize that the effect of the extremely flat topography, coupled with seasonal disconnectivity and the lack of turbulent flow over or through the more sediment‐based dams, created more oxygen‐depleted and redox‐stratified conditions. Previous research has shown that decreasing discharge in spring, before streamflow becomes more intermittent in DMC, drives gradual deoxygenation, with parts of the lower catchment turning frequently hypoxic during low flow periods in summer, a pattern which we also observed during the 2024/2025 sampling period (Wang, Tetzlaff, Goldhammer, et al., 2025; Wang, Tetzlaff, Liu, et al., 2025). During these periods of disconnection, denitrification, S reduction, and desorption of P primarily occur at the water–sediment interface, signifying the influence of seasonal hydrological (dis)‐connectivity between the upstream and downstream channel networks, as well as seasonal source limitations (i.e., increased biological uptake of N by surrounding vegetation during summer) (Smith et al., 2021; Wang, Tetzlaff, Birkel, et al., 2025; Wu et al., 2022). Therefore, we would argue that biogeochemical cycling in the DMC is unlikely to be unilaterally driven by beaver activity, but rather a consequence of beaver‐enhanced mixing of groundwater and surface water in wetland areas, as well as local agricultural underdrainage from saturated soils in winter (Smith et al., 2020; Wang, Tetzlaff, Birkel, et al., 2025; Wu et al., 2021).

In that context, it remains surprising that NO_3_‐N concentrations are reduced so efficiently in SOFL, as denitrification usually requires low‐oxygen/anaerobic conditions (Murray et al., 2023). One possible explanation might be that in beaver ponds, complex environments with zones of low and high oxygen coexist, whereby oxygen‐poor muddy sediments at the bottom of beaver ponds promote denitrification. This would suggest that different geomorphic units may be present within SOFL beaver ponds, with some areas acting as N‐sinks (Murray et al., 2023), while outflowing water is oxygenated through surface oxygenation across beaver dam cascades. Additionally, it is also likely that shallow groundwater in the lower catchment mixing with stream water contributes to diluting NO_3_‐N. One caveat to this hypothesis at this point is that we do not have information on pond geomorphology or oxygen levels directly within beaver ponds. To further test this hypothesis and the diverging DO patterns, additional sampling of N in sediments within beaver ponds and below beaver dams, as well as pond oxygen profiles, and above and below beaver dams, could provide deeper insights into biogeochemical processing capacity and oxygenation within different beaver impoundments. Analysis of N concentrations in local groundwater could also shed additional light on groundwater–surface water interactions and nutrient processing.

### Implications for beaver reintroduction in agricultural catchments

Based on previous research in the DMC catchment on the role of beavers (Smith et al., 2020; Wang, Tetzlaff, Birkel, et al., 2025) and considering the recent decline in beaver activity, we conclude that in intermittent lowland systems like DMC, the presence of beaver dams and effects on water quality are not as straightforward as expected. Besides the hydro‐geomorphic context, the extent of beaver impacts on water and nutrient fluxes, residence times, local water budgets, and biodiversity are also strongly influenced by the length of time beavers remain in a given area (Larsen et al., 2021; Wohl, 2019). As the impacts of beaver dams change over time as they age, are abandoned, or are removed, this may be an important aspect in both catchments. In DMC, we observed occasional dam removal in the upper and lower wetlands, where dams are removed to reduce flooding of nearby fields and infrastructure. While we do not have detailed information on the frequency of dam removal, it appears likely that the lack of fixed long‐term structures like those in SOFL could have an impact on long‐term biogeochemical cycling.

Nevertheless, the persistent presence of beaver for ~20 years in the upstream riparian wetland in DMC has led to increasing and more stable groundwater levels. In this way, the continued presence of beavers in the rehabilitated wetland (including side channels) did increase local surface water leakage to groundwater, as well as residence times downstream of beaver dams, even though dams were occasionally removed in the main channel (Smith et al., 2020). Similarly, at the stream outlet (BB), beaver dams are frequently removed to avoid flooding of a nearby road. However, dams are repeatedly rebuilt in the area, suggesting that the habitat remains attractive to beavers. This is likely due to the strong hydrologic connectivity and flow permanence, and sufficient forage in the lower catchment. An important caveat to our interpretation is that we do not have quantifiable evidence of dam removals; thus, we cannot empirically link spatiotemporal patterns of human interventions with beaver engineering. In the future, it could be of interest to collect targeted information on human involvement in the area, in order to gauge perceptions and evaluate the social dimensions of beaver reintroduction and potential areas of conflict (Auster et al., 2021; Coz & Young, 2020).

Interests between forest and agricultural landowners and beavers remain conflict‐laden in many areas where beavers are reintroduced (Auster et al., 2020; Coz & Young, 2020; Hohm et al., 2024). Even though many of these reintroductions have occurred naturally without much planning, perceptions and coexistence remain challenging (Hohm et al., 2024). In SOFL, a recent decline in the water quality of Lake Schermuetzelsee, into which the stream discharges, sparked local concern about beavers potentially causing deoxygenation and increased influx of P and N into the lake. While the recent expansion of beavers in the SOFL catchment in the last decade seemingly co‐occurred with the lake water quality deterioration, recent analyses of lake sediments have revealed that rather older legacy pollution from former effluent discharge, upstream fertilizer‐based inputs into SOFL, and dewatering of peatlands before beaver re‐colonization have contributed to significant accumulation of nutrients in lake sediments (Meisel, 2025) that may now have been released also due to climate‐related shifts in lake temperature and mixing regimes.

Contrary to those initial assumptions, our data suggest that nutrient concentrations currently de facto decrease with flow in SOFL (Figure 6). Furthermore, analyses of long‐term discharge and nutrient concentrations by the LfU Brandenburg actually showed that as discharge from SOFL declined in the last decade—a side effect of flow attenuation due to beaver dam cascades—also nutrient loads into the lake declined. These findings align with the results of this study, further underlining how beaver dams have enhanced biogeochemical processing in the SOFL. In the context of beaver management, such erroneous assumptions of negative beaver impacts are common and highlight the existence of pervasive skepticism of some stakeholders regarding beaver reintroductions. Based on the example of the SOFL, we would argue for the importance of holistic landscape‐dependent contextualization in environmental assessments to determine whether beaver reintroductions are suitable and can be sustainably managed for long‐term success (Hohm et al., 2024). Especially in agriculturally dominated catchments, there is value in using beaver ponds to manage N‐related diffuse pollution problems (Brazier et al., 2021; Lazar et al., 2015). Nevertheless, stakeholder involvement remains crucial when considering planned reintroductions to limit beaver‐human conflicts and ensure long‐term coexistence.

The differential and highly complex effects of beaver dams in agricultural catchments we report here may run counter to the expectation that we can generalize from one catchment to another. Considering the different landscape settings of both catchments in terms of impacts of beavers on stream water quality, these are not unequivocally clear in DMC. Limited by hydrological stress, wetland size, and management, beaver impacts on biogeochemical processes were less pronounced, emphasizing that these impacts cannot be separated from the wider landscape and climate context in which they occur (Larsen et al., 2021). Especially when considering beavers as a nature‐based solution in agricultural catchments, potential limitations in terms of hydrologic and ecological limits must be considered. This may also be the case in other catchments, where environmental and anthropogenic stressors create differential boundary conditions for hydrologic and ecological functioning. In this context, our study provided a new perspective on how catchment‐scale geomorphology, other physiographic characteristics, and hydrologic connectivity play an important role in the extent to which beaver‐induced habitat engineering and landscape carrying capacity may be constrained in lowland agricultural landscapes. Here, landscape capacity models could be useful to assess limits and potentials of riverscapes to support beaver activities based on water sources, riparian vegetation density, and topography, with additional decision support tools to limit human‐beaver conflicts (Macfarlane et al., 2014, 2017).

## CONCLUSION

We studied two topographically distinct lowland agricultural headwater catchments in the NE of Germany. Both catchments—the SOFL and DMC—are situated in Brandenburg in Eastern Germany, a region that is increasingly drought‐sensitive. Based on the analysis of various catchment settings, we found significant differences in spatiotemporal patterns of beaver distribution in both catchments. Furthermore, physical differences in dam structure and size could be linked to landscape settings (i.e., slope), surface‐groundwater connectivity, and stream characteristics (straightened vs. meandering). In SOFL, an extensive beaver dam cascade system in the lower catchment area had a significant impact on downstream water chemistry, indicating significant denitrification and reduction in organic carbon and fertilizer‐based nutrients. Furthermore, surface‐groundwater connectivity indicates a strong impact on groundwater recharge and drought resilience, due to increased water retention and storage in beaver ponds. In contrast, lower dam density and spatial dynamics of nutrient fluxes in DMC revealed more localized mobilization processes and responses of solutes to varying hydrological connectivity, which could not be linked to beaver dams alone.

Our findings clearly demonstrated how landscape settings, topography, land use, and hydrological boundary conditions play a significant role in the distribution and settlement of beavers, which in turn results in differential and complex effects on local water quality and retention. This suggests that beaver‐mediated impacts on catchments are not uniform across landscapes, warranting consideration of the wider hydro‐geomorphic landscape context for evaluating the potential of beaver‐driven ecosystem engineering and restoration. Ecological restoration of agricultural headwater streams through beaver recolonization or use of beaver dam equivalents as nature‐based solutions offers opportunities to link catchment, larger river, and wetland restoration. Still, open questions remain regarding the impacts of exacerbated global change on terrestrial and aquatic ecosystems and their ability to adapt.

## AUTHOR CONTRIBUTIONS

*Conceptualization*: Maria Magdalena Warter, Dörthe Tetzlaff, and Chris Soulsby. *Methodology*: Maria Magdalena Warter, Dörthe Tetzlaff, and Chris Soulsby. *Investigation*: Maria Magdalena Warter, Lisa Grof, Luisa Wurbs, and Tobias Goldhammer. *Formal analysis*: Maria Magdalena Warter. *Data curation*: Maria Magdalena Warter, Lisa Grof, and Luisa Wurbs. *Writing–original draft*: Maria Magdalena Warter. *Writing—review and editing*: Maria Magdalena Warter, Dörthe Tetzlaff, Chris Soulsby, and Tobias Goldhammer. *Visualization*: Maria Magdalena Warter. *Supervision*: Dörthe Tetzlaff and Chris Soulsby. *Funding acquisition*: Maria Magdalena Warter and Dörthe Tetzlaff.

## CONFLICT OF INTEREST STATEMENT

The authors declare no conflicts of interest.

## Supporting information


Appendix S1.

## Data Availability

Datasets are available in the Leibniz‐Institute of Freshwater Ecology and Inland Fisheries (IGB) Freshwater Research and Environmental Database (FRED) database as follows: Sophienfließ dataset (Dämpfling, 2026b), https://doi.org/10.18728/igb-fred-1150.2; Demnitz Mill Creek dataset (Dämpfling, 2026a), https://doi.org/10.18728/igb-fred-1149.2; beaver dam dataset (Warter, 2026), https://doi.org/10.18728/igb-fred-1129.1.
