# Supplementary material for: Catchment structure and functioning mediates beaver activity in contrasting lowland agricultural landscapes
Source: Ecol Appl. 2026 Oct 26;36(7):e70335. doi: 10.1002/eap.70335 (PMC13710877; doi:10.1002/eap.70335)
Supplement: Supplementary file 1 [file EAP-36-e70335-s001.pdf]

Appendix S1

Catchment structure and functioning mediates beaver activity in contrasting lowland agricultural landscapes

Maria Magdalena Warter, Dörthe Tetzlaff, Tobias Goldhammer, Luisa Wurbs, Lisa Grof, Franziska Schmid, and Chris Soulsby

Ecological Applications

- **Table S1:** Summary of measured parameters, including hydroclimate, discharge, water chemistry, water quality and beaver activity in each catchment.
- **Table S2:** Sub-catchment areas for main sample points in the Sophienfliess and Demnitzer Mill Creek catchment.
- **Figure S1:** Time series of daily precipitation and precipitation isotopes, and daily streamflow and stream water isotopes at Sophienfliess and Demnitzer Mill Creek.
- **Figure S2:** Groundwater quality of sampled groundwater inflows into the lower Sophienfliess catchment from most upstream to most downstream sampling point for the 2024-25 sampling period.
- **Figure S3:** Boxplots of nutrient and DOC concentrations for the period January 2024-December 2025 in the SOFL (green, left) and DMC (blue, right) from upstream to downstream. Note the differences in magnitude on y-axis for NO_3N, DOC and SO_4.
- **Figure S4:** Timeseries of SRP, NO_3N, DOC and SO_4 concentrations for the period January 2024 – December 2025 in the Sophienfliess (green, left) and Demnitzer Mill Creek (blue, right) for each sample location.
- **Table S3:** Damping ratios (DR) at each stream sample location in the Sophienfliess and Demnitzer Mill Creek.
- **Table S4:** Summary of linear mixed model results for the Sophienfliess and Demnitzer Mill Creek indicating positive (↑) or negative (↓) effects and significance levels at the *0.05, **0.01 and ***0.001 level.

Table S1: *Summary of measured parameters, including hydroclimate, discharge, water chemistry, water quality and beaver activity in each catchment.*

Catchment	Climate	Discharge	Water Chemistry	Water Quality	Beaver activity
**SOFL**	Precipitation (hourly)<br>Temperature (hourly)	S10 (outlet) (15-min) 2019-February 2025	10 locations<br>Bi-weekly (Feb 2024-April 2024)<br>Monthly (Apr 2024-Dec 2025)	10 locations<br>Bi-weekly (Feb 2024-April 2024)<br>Monthly (Apr 2024-Dec 2025)	Spatial survey 2024, 2025
**DMC**	Precipitation (15-min)<br>Temperature (hourly)	Peat North (15min) Bruch Mill (15min) Demnitz Mill (15min) 2019-2025	5 Locations Weekly (2019-2025)	5 Locations Weekly (2019-2025)	Spatial surveys since 2012

Table S2: *Sub-catchment areas (in km^2) for main sample points in the Sophienfliess and Demnitzer Mill Creek.*

**Sophienfliess**		**Demnitzer Mill Creek**	
Sub-catchment area (km^2)		*Sub-catchment area (km^2)*	
S00	11.24	Peat North (PN)	18.5
S01	22.01	Peat South (PS)	21.3
S02	23.76	Peat Ditch (PD)	25.7
S04	34.8	Bruch Mill (BM)	40.7
S05	35.7	Demnitz Mill (DM)	43.6
S06	36.3	Berkenbrueck (BB)	66.4
S08	37.1		
S09	37.2		
S10	39.1		

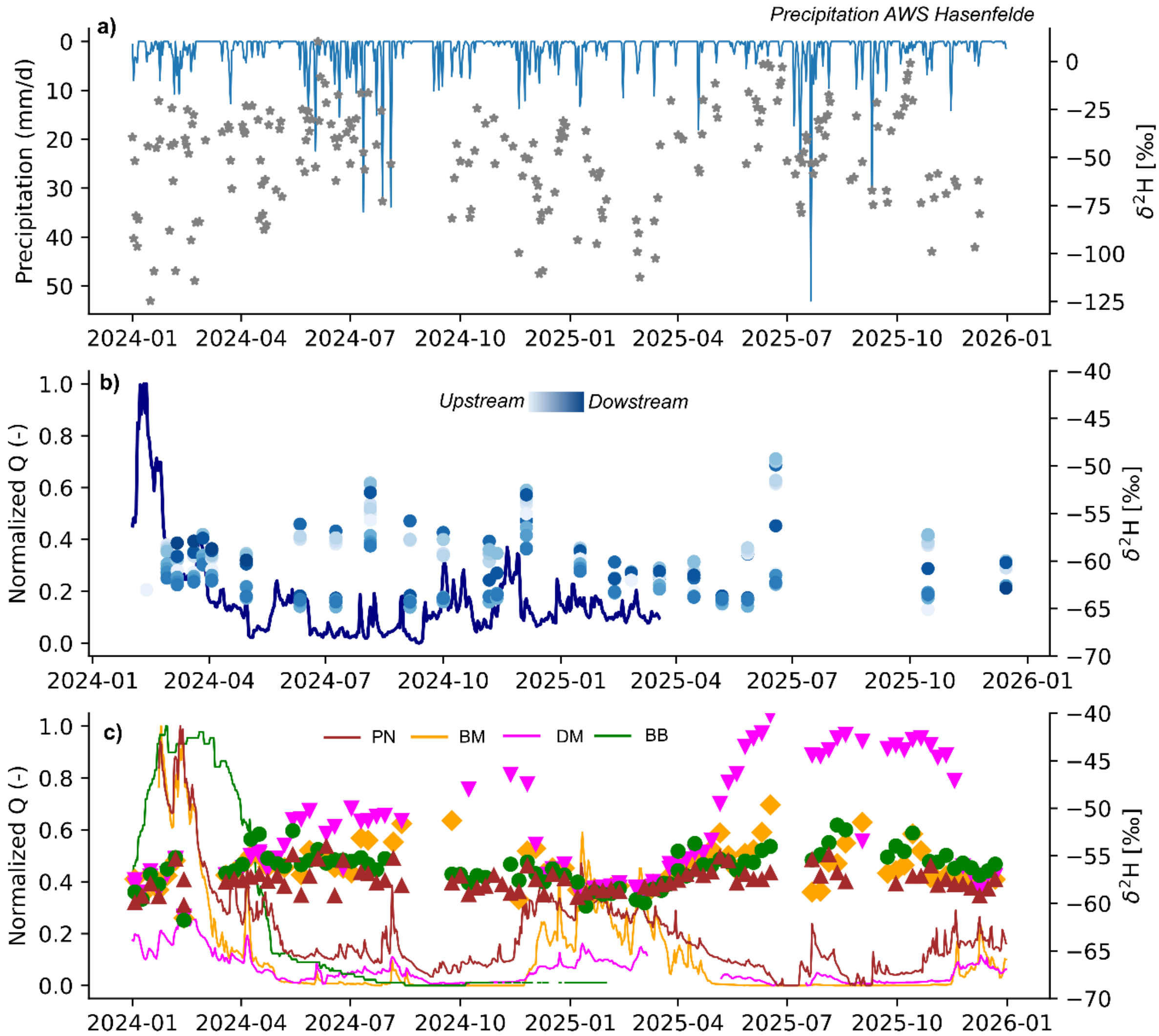

Figure S1: *a) Daily precipitation (in mm/d) and precipitation isotopes (δ^2H in ‰) from the AWS Hasenfelde near the Demnitzer Mill Creek for the sample period 2024-2025. Normalized stream discharge (from daily specific discharge) with stream water isotopic composition for the b) Sophienfliess (2024 – February 2025) and c) selected sample sites in the Demnitzer Mill Creek 2024-2025. Several data gaps in discharge exist in DMC.*

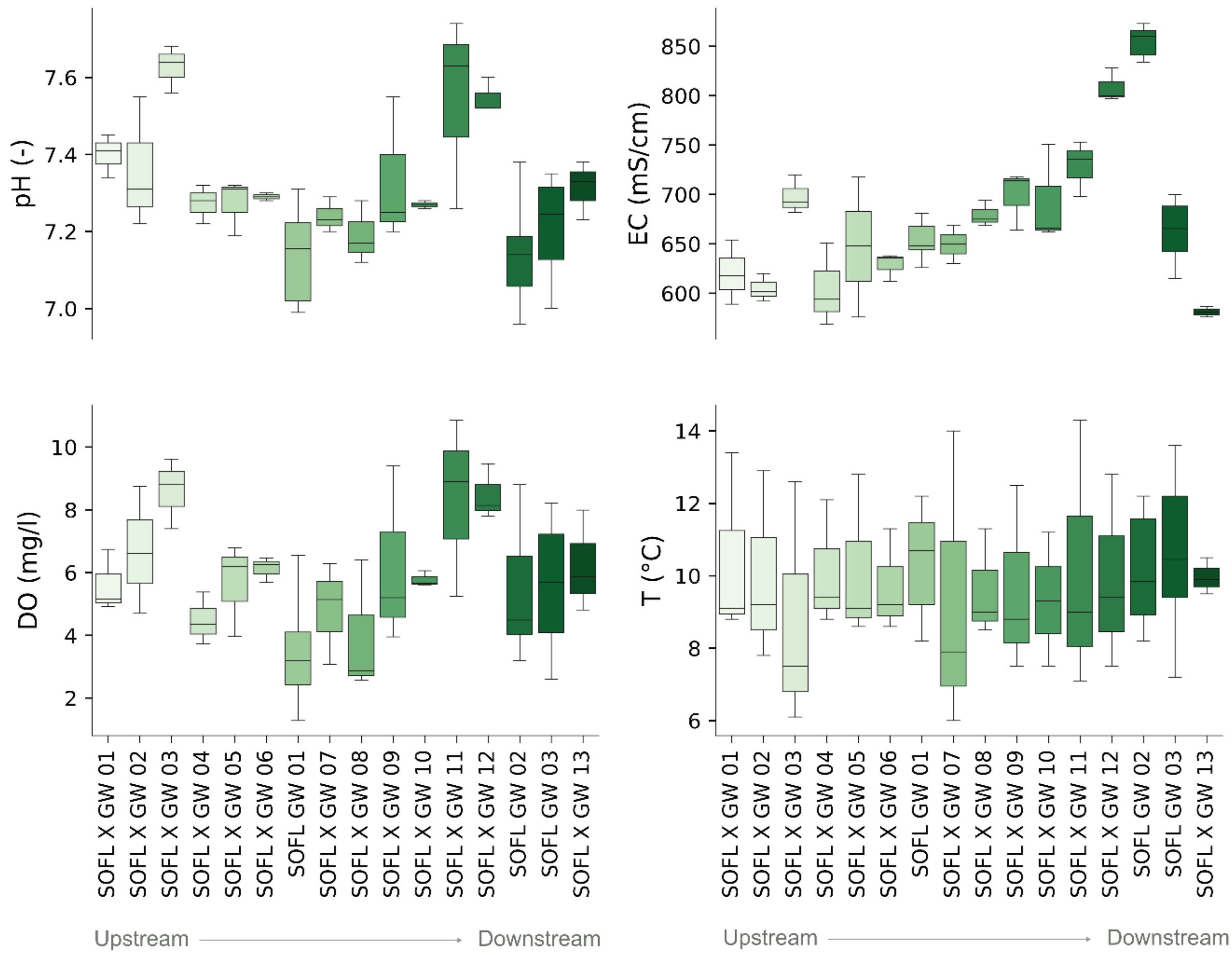

Figure S2: *Groundwater quality of sampled groundwater inflows into the lower Sophienfliess catchment from most upstream to most downstream sampling point for the 2024-2025 sampling period.*

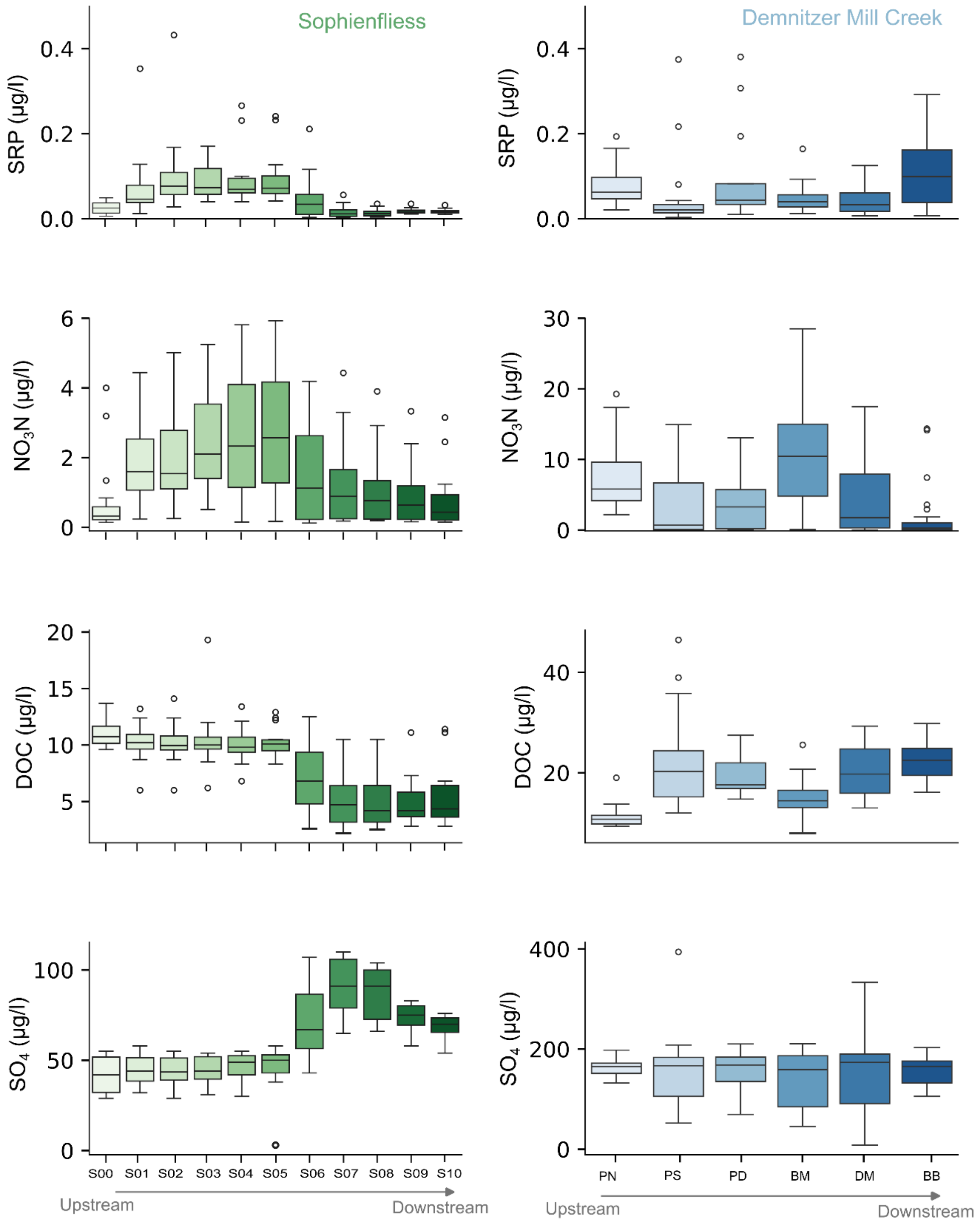

Figure S3: *Boxplots of nutrient and DOC concentrations for the period January 2024-December 2025 in the SOFL (green, left) and DMC (blue, right) from upstream to downstream. Note the differences in magnitude on y-axis for NO_3N, DOC and SO_4.*

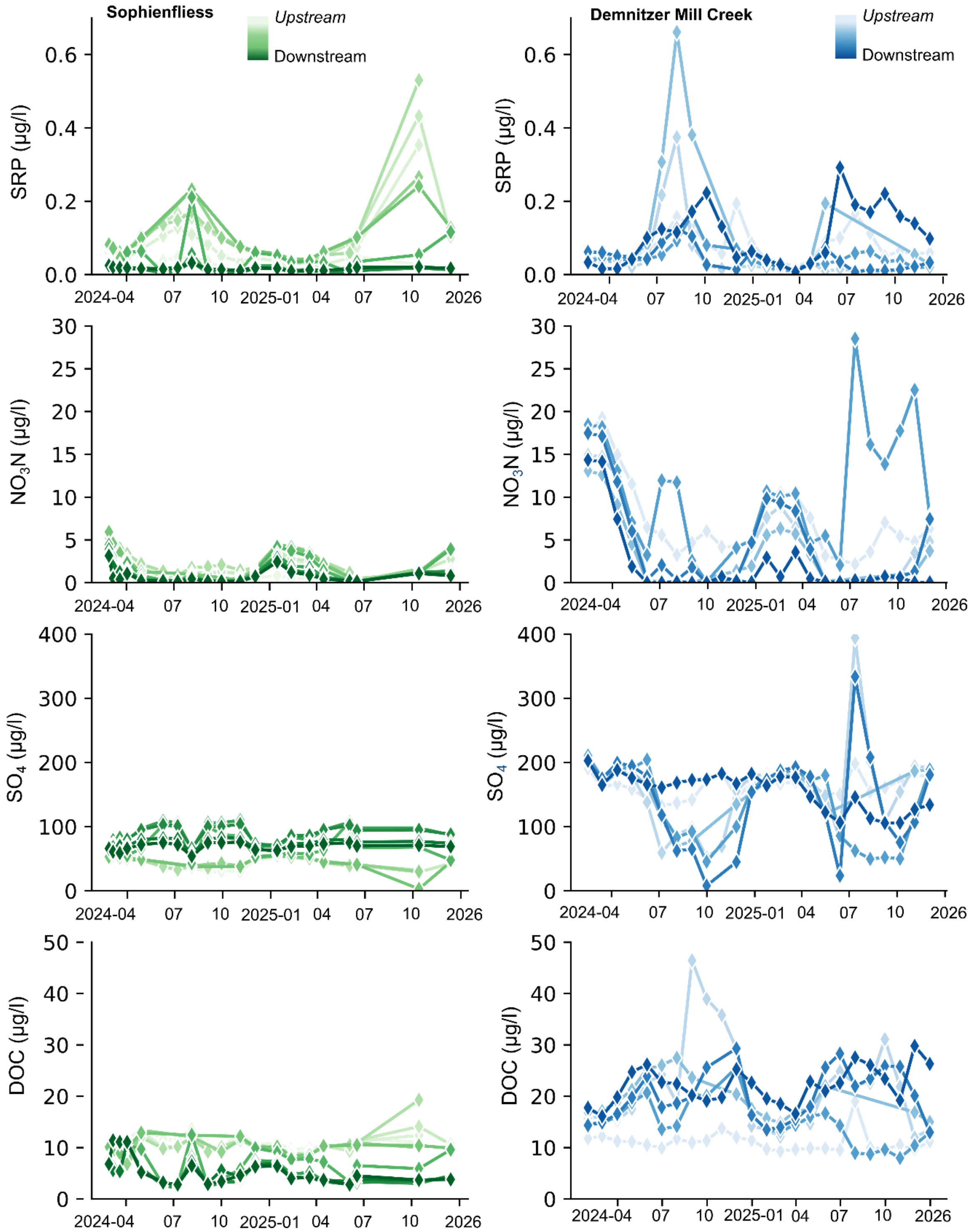

Figure S4: *Timeseries of SRP, NO_3N, DOC and SO_4 concentrations for the period January 2024 – December 2025 in the Sophienfliess (green, left) and Demnitzer Mill Creek (blue, right) for each sample location.*

Table S3: *Damping ratios (DR) at each stream sample location in the Sophienfliess and Demnitzer Mill Creek.*

**Sophienfliess**		**Demnitzer Mill Creek**	
Site ID	*$\delta^{18}O$ DR*	*Site ID*	*$\delta^{18}O$ DR*
S00	0.13	Peat North	0.24
S01	0.11	Peat South	0.86
S02	0.12	Peat Ditch	1.0
S04	0.13	Bruch Mill	0.38
S05	0.14	Demnitz Mill	0.65
S06	0.14	Berkenbrueck	0.14
S08	0.09		
S09	0.07		
S10	0.07		

Table S4: *Summary of linear mixed model results for the Sophienfliess and Demnitzer Mill Creek indicating positive (↑) or negative (↓) effects and significance levels at the *0.05, **0.01 and ***0.001 level.*

**Predictor**	***DO***	**SRP**	**NO_3N**	**DOC**	**SO_4**
Sophienfliess					
Dam density	↓ ***	↓ ***	↓**	↑ ***	↓ *
Slope	↓ ns	↓ **	↓ **	↑ **	↑ **
Groundwater (Ca)	↓ ***	↓ ***	↑ ns	↓ ***	↑ ***
Temperature	↓ ***	↑ ***	↑ ***	↑ ***	↓ ns
pH	↑ ***	↓ ns	↑ ns	↑ **	↓ *
Landuse	↓ ns	↓ ns	↑ ns	↑ **	↓ ns
Demnitzer Mill Creek					
Dam density	↑ns	↓ **	↓ **	↑ ns	↑ ns
Slope	↓ *	↑ *	↓ ns	↑ ns	↑ ns
Groundwater (Ca)	↓ ns	↓ ***	↓ ***	↑ ***	↑ ***
Temperature	↓ **	↑*	↓ ns	↑ ***	↓ ns
pH	↑***	↑ns	↑**	↓ ns	↓ ns
Landuse	↑ns	↑ns	↓ ns	↑ *	↓ ns
